# Interplay between disinfection and the enigmatic diplomonad parasite *Spironucleus salmonicida* in Atlantic salmon

**DOI:** 10.1038/s41598-026-51626-4

**Published:** 2026-05-08

**Authors:** Carlo C. Lazado, Hanne Brenne, Didrik Olaisen, Gunhild S. Johansson, Miroslava Hansen, Bjørn-Steinar Saether, Jelena Kolarevic, Gerrit Timmerhaus, Torstein Tengs, Lill-Heidi Johansen

**Affiliations:** 1 The Norwegian Institute of Food, Fisheries and Aquaculture Research, Osloveien 1, Nofima, Ås, 1433 Norway; 2 The Norwegian Institute of Food, Fisheries and Aquaculture Research, Muninbakken 9-13, Nofima, Tromsø, 9019 Norway; 3 UiT – The Arctic University of Norway, Universitetsvegen 40, Tromsø, 9019 Norway

**Keywords:** Aquaculture, Biosecurity, Microbiota, Mucosal health, Ozone, RAS, Spironucleosis, UV, Diseases, Microbiology

## Abstract

The diplomonad parasite *Spironucleus salmonicida* causes spironucleosis, a systemic infection characterised by internal haemorrhaging, splenomegaly, and granulomatous lesions in multiple organs. Prevention is the primary control strategy, particularly in recirculating aquaculture systems (RAS). This study evaluated the effectiveness of common water-disinfection methods against the parasite. In situ experiments assessed parasite survival following exposure to low- and medium-pressure ultraviolet (UV) radiation at different doses. A subsequent in vivo trial simulated a biosecurity breach in RAS using Atlantic salmon smolts. Two loop-disinfection strategies were tested: continuous UV treatment (70–80 mJ/cm²) and ozone (300–350 mV oxidation–reduction potential), alongside a no-disinfection control. The parasite was introduced via make-up water over three days, and fish were monitored for four weeks. In situ, medium‑pressure UV doses ≥ 50 mJ/cm² immediately inactivated the parasite, whereas lower doses achieved inactivation within 48 h. In vivo, no fish developed disease, and the parasite was not detected in any group. Disinfection did not affect mucosal integrity, although ozone increased expression of oxidative stress markers in the gills. UV and ozone also altered microbial community composition. Overall, *S. salmonicida* was sensitive to medium-pressure UV, and under the tested conditions did not persist or cause disease in RAS.

**Supplementary Information:**

The online version contains supplementary material available at 10.1038/s41598-026-51626-4.

## Introduction

The diplomonads (classified as Fornicata in the supergroup Excavata) are a group of anaerobic flagellates that have two diploid nuclei^1^. Within this group, *Spironucleus* species have attracted significant interest due to the serious diseases they can cause in both companion and farmed animals^2–4^. In salmonids, *Spironucleus salmonicida* (formerly known *S. barkhanus*) is known to cause systemic infections with pathologies characterised by internal haemorrhaging, splenomegaly, and granulomatous lesions in the kidneys, liver, spleen, and muscles^5^. Under controlled laboratory conditions, trophozoites have been detected in the gut, faeces, skin, and gills of experimentally infected fish and are believed to multiply asexually in the intestine, breach the mucosal barrier, and spread systemically via the bloodstream^6–8^.

Recurrent *S. salmonicida* infections are often referred to as the “salmon killer” due to the high mortality risk once the disease is established. Transmission is highly efficient, especially under intensive aquaculture conditions, which suggests that stress related to husbandry practices may be a contributing factor^7–9^. Metronidazole has been shown to be an effective treatment against spironucleosis, but it has been banned in Europe since the late 1990 s due to its carcinogenic properties^10,11^. Currently, no treatment for spironucleosis is available, leaving enhanced biosecurity measures as the only effective preventive strategy on farms.

Disinfection is a key strategy for preventing the entry and spread of pathogens in aquaculture systems and plays a crucial role in ensuring high biosecurity on fish farms^12^. In particular, ultraviolet (UV) irradiation in the UVC spectral range is used for inlet water, recirculating water, and wastewater in land-based aquaculture systems^13^. This strategy is effective against a broad range of pathogens and is relatively safe, as no disinfection by-products or residuals that may be detrimental to fish have been identified^14,15^. UVC irradiation inactivates microorganisms primarily by inducing photochemical reactions that damage their DNA or RNA and specifically forming pyrimidine dimers that disrupt genetic replication and transcription. Therefore, using UV does not necessarily kill microorganisms during the disinfection process; rather, it inactivates them, leading to a loss of microbial functions^16^.

The most effective inactivation occurs at wavelengths between 250 and 270 nm, which is a range of energy that is strongly absorbed by the nucleic acids of microorganisms^17^. Two types of UVC lamps are currently used in aquaculture: low-pressure (LP) and medium-pressure (MP) lamps. LP lamps are the most commonly used and emit nearly all their energy at a single wavelength (254 nm), which makes them monochromatic and effective for microbial inactivation. On the other hand, MP lamps emit a broad polychromatic spectrum ranging from 220 to 300 nm, which allows for high energy output in a compact design and enhances disinfection effectiveness by damaging enzymes and other DNA-repair mechanisms, leading to irreversible harm to microorganisms^18^.

Previous studies indicate that trophozoites of *Giardia intestinalis*, a parasite closely related to *S. salmonicida* can survive relatively high UV doses but may exhibit reduced growth at low exposure levels, with delayed growth reported at doses as low as 1–2 mJ/cm². In contrast, cysts are more sensitive to UV exposure, with doses around 10 mJ/cm² preventing the establishment of viable trophozoites despite excystation^19^. For *S. salmonicida*, no cyst stage has yet been confirmed, although it is assumed that this parasite also goes through such a stage during its life cycle, as shown for similar parasites^9,20^. Therefore, it is assumed that effective inactivation of *S. salmonicida* flagellates may require UV doses comparable to those needed for *G. intestinalis*.

Additional disinfection methods, such as ozonation, may also be necessary^21^. Disinfection using ozone primarily occurs through oxidation or peroxidation of biomolecules either directly or indirectly via free-radical reactions, which are considered its main mechanisms of action^22^. Although ozone is highly effective against a wide range of pathogens, it is an unstable molecule that quickly breaks down in water, and if not carefully managed, residual ozone in the water can be harmful or even lethal to the fish. This risk is especially high in saline systems, where more persistent and potentially toxic by-products such as free bromine and bromoamines are more likely to form^23,24^.

The majority of land-based production of salmon employs recirculating aquaculture systems (RAS). Although biosecurity measures for these systems have improved in recent years, multiple disease outbreaks have still been reported^12,25^. Due to long water retention, high nutrient levels, and dense stocking, fish pathogens can accumulate in RAS water or biofilms^21^. Using RAS loop disinfection methods, such as UV and ozone, enhances water quality and helps control the spread of pathogens in the event of a biosecurity breach.

The last spironucleosis outbreak in Northern Norway occurred in 2022–2023 during the grow-out stage at sea^25^. There is limited knowledge about how *S. salmonicida* survive and potentially establish themselves in biofilters and bioreactors in RAS^25^. However, a likely hypothesis is that the parasite enters the RAS facility through the intake water^26^, after which the fish may carry the parasite until it is transferred to the sea.

We investigated the effects of UV treatment on the survival of *S. salmonicida* in simple controlled systems in situ and in a more complex environment in vivo using experimental RAS units with Atlantic salmon smolts. We also compared the effectiveness of UV disinfection with ozone in vivo. A biosecurity breach was simulated in systems with and without RAS loop disinfection to evaluate the combined effects of disinfection and pathogen breach on the fish and the systems.

## Materials and methods

### Ethics statement

All experimental procedures involving fish were conducted in compliance with institutional guidelines, the ARRIVE reporting standards, and the EU Directive 2010/63/EU. The study was approved by the Norwegian Food Safety Authority (FOTS 30459). Fish handling and sampling procedures were designed to minimise stress, and all efforts were made to ensure good animal welfare and humane conditions throughout the experiments.

### Origin and routine culture conditions for *S. salmonicida*

The parasite used in the trial was isolated from an infected Atlantic salmon smolt from a commercial sea cage in Northern Norway, which was delivered to Nofima in Tromsø in the autumn of 2022. The parasite was isolated from the heart of a diseased fish exhibiting excessive scale loss, superficial wounds with bleeding, severe internal haemorrhaging, hepatomegaly with nodulations, and splenomegaly. It was subsequently cultured in Keister’s Complete Medium at 12 °C. The identity of the isolated parasite was verified by real-time quantitative PCR (RT-qPCR), and its morphology was observed using light microscopy.

**Fig. 1:**
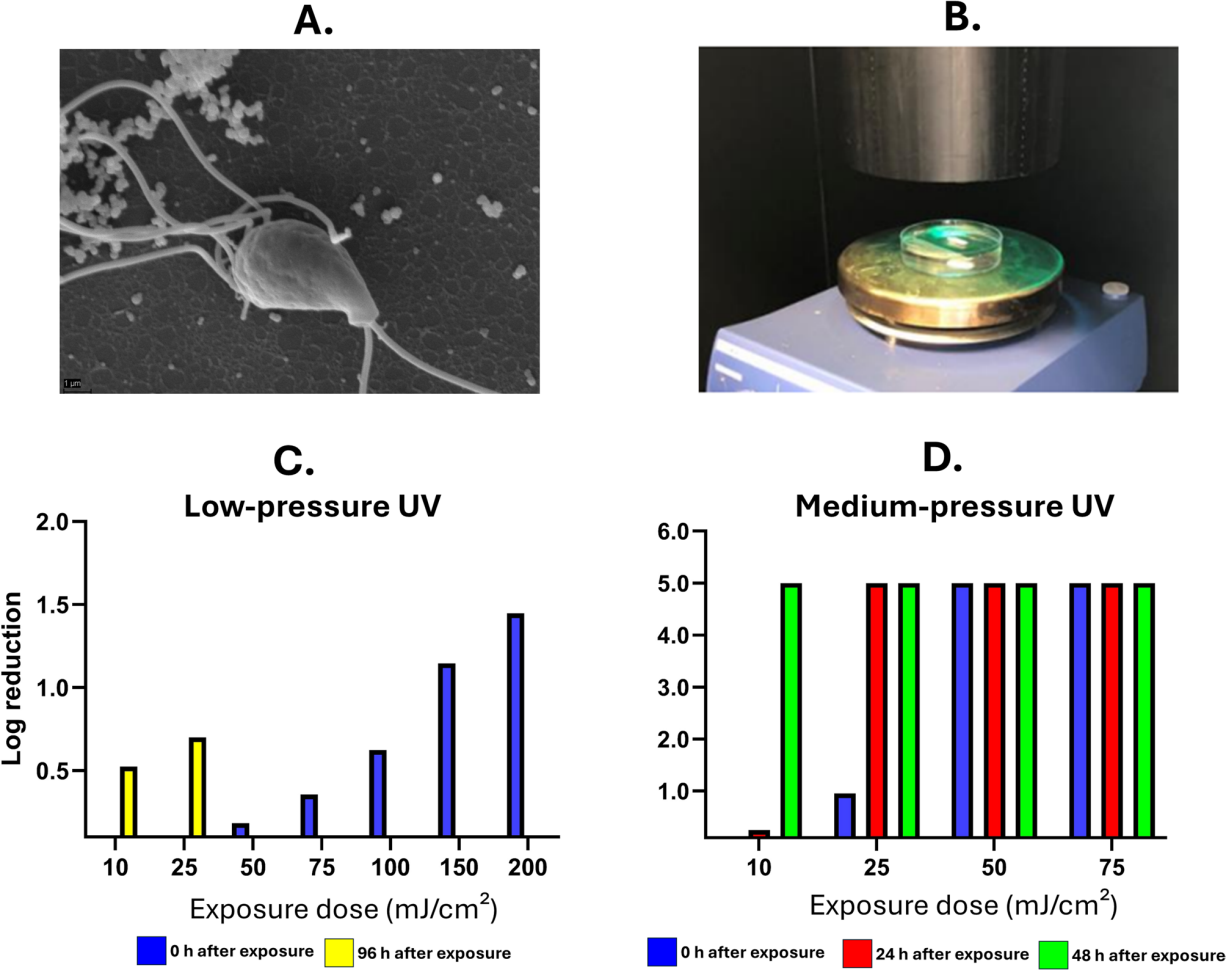
In situ UV exposure of *S. salmonicida*. (**A**) A representative SEM image of the parasite. Note the 8 flagella: 6 emerging from the anterior part and 2 flagella at the posterior end. (**B**) Exposure setup used for in situ UV dose-response trial. Note that the parasite was suspended in solution during exposure. Log reduction in the viable counts of the parasite after progressive exposure to increasing UV under either (**C**) low-pressure or (**D**) medium-pressure lamps. Viability was checked right after exposure and at 24, 48, and 96 h after exposure. (**C**) and (**D**): Data are presented as log reductions calculated from the mean of three replicate counts at Time 0 and six replicate counts at 24, 48, and 96 h after exposure. For Medium-pressure UV, each test was repeated three times. Only minimal variation was observed among replicates. Log reduction provides a logarithmic measure of disinfection performance, where each 1 log reduction corresponds to a 90% decrease in viable microorganisms, and higher values indicate progressively greater inactivation (e.g. 5 log reduction equals 99.999% reduction).

A parasite culture was also sent to the microscopy lab at the Imaging Centre of the Norwegian University of Life Sciences for Scanning Electron Microscopy (Fig. 1A). The parasite was routinely cultured in Keister’s Complete Medium and passaged every 5 days by transferring a 1.5-ml aliquot into a fresh 15-ml tube, which was then filled completely with fresh Keister’s Complete Medium under anaerobic conditions. The culture was incubated at 12 °C.

### Trial 1: UV exposure in situ

In situ bench-scale experiments were conducted to determine the minimum UV doses required to inactivate 99.9% of *S. salmonicida*. LP and MP UV lamps were evaluated. The experimental setup used two collimated beam apparatuses (Atlantium Technologies), as shown in Fig. 1B. Fresh cultures of the parasite were washed three times with 0.3% sodium chloride (NaCl) by centrifugation at 700 *g* for 5 min and then resuspended in 0.3% (w/v) NaCl to achieve UV transmission at 254 nm (UVT_254_) greater than 70%. This brackish water condition was necessary to maintain parasite survival as the organism was found to die almost immediately in freshwater or with salinity greater than 12 ppt.

Washed cultures with a volume of 20 mL that contained approximately 10^5^ parasites were used in the UV exposure trials. The effect of UV radiation on parasite survival was assessed using culture-viability tests and microscopy, including counts of live and dead cells. Washed parasites were transferred to a Petri dish and exposed to the specified UV dose. For LP UV, the UVT_254_ value was entered into the Atlantium calculator to determine the exposure time needed to reach the desired dose. The parasites were progressively exposed to doses of 0, 10, 25, 50, 75, 100, 150, and 200 mJ/cm². For MP UV, the absorbance was measured at 230–300 nm, converted to transmission, and entered into the same calculator to determine the exposure time. Doses of 0, 10, 25, 50, and 75 mJ/cm² were tested.

Parasite motility was recorded before and after exposure at each dose level. To evaluate the effect on viability, 300 µL of culture was sampled after each exposure. The samples were stained with eosin Y and nigrosine and counted using a Burker counting chamber. Parasite viability was determined based on membrane integrity: viable parasites excluded eosin Y, while non‑viable parasites stained positive. Nigrosine provided background contrast to facilitate discrimination between live and dead cells. After the highest UV exposure dose for both LP and MP treatments, 100 µL of culture was transferred to a glass tube with 250 µL of Keister’s Complete Medium. The tube was sealed with parafilm and incubated at 12 °C. For the group exposed to LP UV, live and dead cell counting and microscopy were performed 96 h after exposure, while similar assessments were performed at 24 and 48 h after exposure in the MP UV group.

### Trial 2: in vivo biosecurity breach

A fish trial was conducted at the Tromsø Aquaculture Station using nine individual RAS units, as previously described^27^. Briefly, each modular unit consists of a cylindro-conical fish tank (V = 0.5 m^3^) that is connected to a water treatment system. Water filtration and treatment were performed using a drum filter (micro-screen mesh size = 40 μm), a moving bed bioreactor (V = 0.2 m^3^, 750 m^2^/m^3^ bio-media), a gas exchange unit (CO_2_-degasser cylinder), a foam fractionator, a pressure oxygen cone (0.6 Bar), and a temperature control unit. The total water volume of the RAS was 0.8 m^3^, the water flow to the fish tank was 1 500 L/h, and the fish tank hydraulic retention time was 20 min. An LED light above each unit provided continuous illumination.

**Fig. 2:**
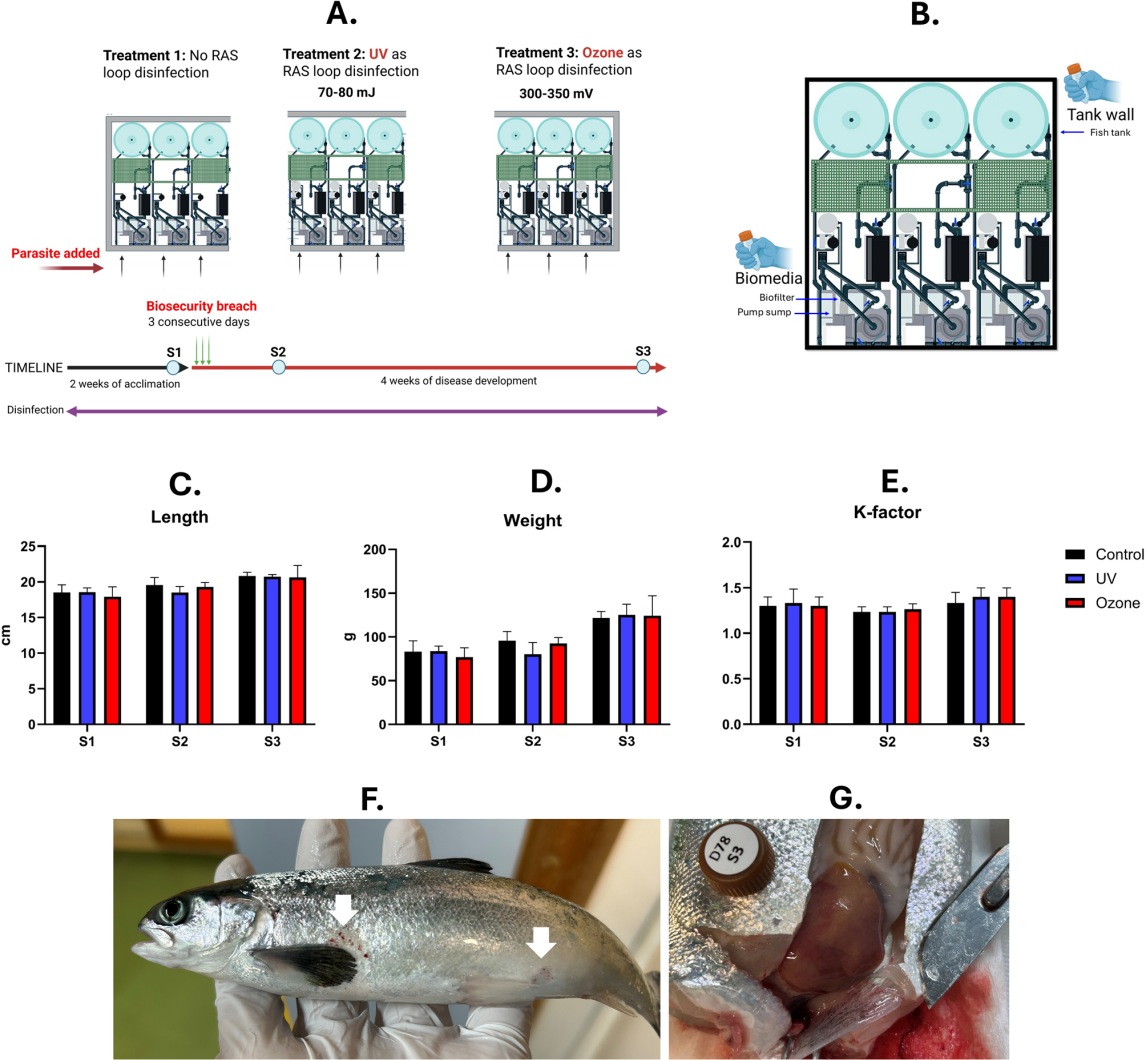
Simulated biosecurity breach of *S. salmonicida* in RAS. (**A**) Experimental set-up and timeline. Each treatment group consisted of three replicate RAS units. Flagellates were introduced via the intake water for three consecutive days, after which disease progression was monitored over a 4-week period. Sampling was conducted at three time points: sampling 1 (S1) took place before the biosecurity breach at 2 weeks after the acclimation period; sampling 2 (S2) occurred 2 weeks after the breach; and sampling 3 (S3) was conducted 4 weeks after the breach at the end of the trial. Swabs were collected from five biomedia and the tank wall at each time point, which coincided with fish sampling. (**B**) Diagram of the RAS unit, which originally appeared in a previous study^27^ and is reproduced here under the Creative Commons BY license. Sampling locations for microbiota collection are highlighted. Additional details on the configuration and components of the experimental RAS are provided in Mota et al.^27^. (**C–E**) Performance parameters measured throughout the trial included length (**C**), weight (**D**), and condition factor or K-factor (**E**). There were 15 fish per treatment group at each time point. No significant differences were observed among the groups for these parameters. (**F–G**) At S3, some fish exhibited gross pathologies such as scale loss and haemorrhaging (**F**, arrow), and liver discolouration (**G**); however, these changes could not be attributed to spironucleosis since the fish were negative for the parasite.

Each RAS unit was stocked with 100 fish with an initial individual weight of approximately 70–80 g (Fig. 2A). Each treatment group consisted of three replicate RAS units. Units 1 to 3 were the control group with no RAS loop disinfection, while units 4 to 6 received UV disinfection using an LP UV unit, which delivered an estimated dose of 70–80 mJ/cm² at 100% UV transmission, partly based on the in situ trial and the operational capacity of the UV units already installed in the system. Units 7 to 9 were treated with ozone after the recirculated water passed through the biofilter, which exposed the fish to an oxidation-reduction potential of 300–350 mV. This range was previously identified as safe for brackish water-adapted Atlantic salmon^23^. A PVC curtain separated each unit to prevent cross-contamination. The trial consisted of two phases: a 2-week acclimation period (phase 1) followed by a 4-week disease-development phase (phase 2). RAS loop disinfection (units 4–9) was applied continuously throughout the entire 6-week trial.

After 2 weeks of acclimation, a biosecurity breach was simulated by introducing a fresh culture of *S. salmonicida* (10,000 flagellates/mL) into the system. They were introduced via the make-up water in the pump sump at a rate of 1% (v/v) of the total daily make-up volume, which was 20 L/day (Fig. 2B). This was carried out for 3 consecutive days. This biosecurity breach model, supported by anecdotal evidence from the industry, assumed that the parasite enters RAS facilities through intake water. The following rearing conditions were maintained throughout the trial: continuous feeding, temperature at 12 °C, salinity at 12 ppt, a 24 L:0D photoperiod, and dissolved oxygen saturation greater than 80%. Several water quality parameters were also controlled according to predefined thresholds. The details of the monitored parameters and variables are provided in **Supplementary File 1**.

### Fish and microbiota sampling

There were three sampling points in total: sampling 1 (S1) was conducted before the biosecurity breach at 2 weeks after the acclimation period, sampling 2 (S2) occurred 2 weeks after the breach, and sampling 3 (S3) took place 4 weeks after the breach at termination. At each time point, fish were fasted overnight, and five fish were randomly selected from each replicate tank. There were 15 fish in each experimental group in total. The fish were euthanised by bath overdose of Finquel Vet (Tricaine Methanesulfonate, 1000 mg/g, MSD Animal Health, Oslo, Norway) at a concentration greater than 135 mg/L.

The weight and length of each sampled fish were measured. These measurements were used to calculate Fulton’s condition factor (K) using the formula, K factor = 100 x weight in g/length^3^. Blood was drawn from the caudal artery, and plasma was separated by centrifugation at 2000 *g* for 10 min and stored at −40 °C until analysis. Tissue samples were collected from the skin, gills, foregut, and hindgut. Skin samples were taken from the area just below the dorsal fin, and the second gill arch was sampled. The entire gut was excised, and any remaining digesta was gently removed. The gut was then rinsed once with chilled 1× phosphate-buffered saline, after which sections of the foregut and hindgut were dissected.

Each biopsy was divided into two portions: one that was immediately placed in 10% buffered formalin (Biopsafe^®^, Vedbæk, Denmark) for histology and a smaller portion that was preserved in RNAlater™ Stabilisation Solution (ThermoFisher Scientific, Dreieich, Germany). After incubation overnight at 4 °C, the RNAlater samples were stored at −40 °C. During the final sampling, peritoneal fluid was obtained from fish exhibiting gross pathologies such as discoloured liver and blood spot patches on the skin, and the sample was suspended in Keister’s Complete Medium. The suspension was incubated at 12 °C and propagated according to the routine procedures described in the in situ exposure trial. Parasite growth was subsequently monitored by microscopy.

Environmental samples for microbiota sequencing were also collected at each sampling point. Five individual biomedia samples were randomly collected from the biofilter using a sterile cup and placed in a ziplock plastic bag. Additionally, a surface swab was taken from a submerged section of the tank wall, approximately 2 inches × 2 inches, using FLOQSwabs^®^ (COPAN, Brescia, Italy) (Fig. 2B). The surface was swabbed 10 times horizontally and vertically, with sampling locations varied across the three time points to avoid repeated collection from the same area. Both sample types were immediately placed on dry ice and later stored at −80 °C until analysis.

### Molecular detection of the parasite

Total DNA was isolated from swabs, plasma, gills, skin, and the two gut sections using an RNAdvance Tissue Total Isolation Kit (Beckman Coulter, Indiana, USA). The presence of the parasite was assessed by RT-qPCR using TaqMan Fast Advanced Master Mix (Applied Biosystems) according to Miller et al.^28^. Reactions were run on a QuantStudio™ 5 Real-Time PCR System. Each reaction volume contained 4 µL of DNA diluted to 1:10, 5 µL of TaqMan Master Mix, 0.5 µL each of forward and reverse primers (final concentration 900 nM), and probes at a final concentration of 250 nM, which targeted the 18 S region of *S. salmonicida* (**Supplementary File 2**). The thermocycling protocol consisted of uracil-DNA glycosylase (UNG) incubation at 50 °C for 2 min, polymerase activation at 95 °C for 10 min, followed by 40 cycles of 95 °C denaturation for 15 s and annealing/extension at 60 °C for 1 min. Additional samples were submitted to an external diagnostic laboratory for independent verification.

### Histological evaluation

Gill and skin samples fixed in formalin were processed for histomorphological evaluation according to the standard protocol at Nofima’s histology laboratory in Ås, Norway. Tissue was processed using an automated tissue processor (LOGOS EVO, Milestone) and embedded in paraffin. Tissue blocks were sectioned at a thickness of 2 μm and stained with Alcian Blue–Periodic Acid Schiff (AB–PAS). The stained sections were then digitised using a Leica APERIO CS2 digital slide scanner (Leica Biosystems, USA).

Scans were uploaded to the Aiforia^®^ platform, and a region of interest (ROI) was drawn on each sample (1 ROI per sample). Each sample was analysed using histomorphometric models that were developed in-house for Atlantic salmon gills and skin based on Aiforia^®^ AI^29^. The Aiforia AI^®^ Skin model was developed using at least 5768 training regions, derived from a minimum of 125 samples across 24 projects. On the other hand, the Aiforia^®^ AI Gill model was developed using at least 3370 training regions derived from 97 samples across 8 projects. Briefly, these AI models have been trained to account for different identified features in a chosen ROI. The output files from Aiforia^®^ were processed in custom R-pipelines that pre-filtered and created datasets for group-wise comparisons. The variables in the gill dataset were normalised to the total lamellar area of the particular sample and presented as a percentage of that area. The skin samples were normalised to the sample’s length in millimetres and presented in µm per mm of skin.

### Gene expression profiling

Immune and stress responses in different mucosal organs were assessed by gene expression analysis. Total RNA was isolated from the gills, skin, foregut, and distal gut using an Agencourt RNAdvance™ Tissue Total RNA Purification Kit. RNA purity and quantity were determined by a NanoDrop 8000 Spectrophotometer (ThermoFischer Scientific, USA). cDNA was generated from 250 ng/µL of normalised RNA using the High-Capacity cDNA Reverse Transcription Kit (Applied Biosystems, CA, USA) in a 20-µL reaction volume. Reverse transcription was carried out in a Veriti™ 96-Well Thermal Cycler (Applied Biosystems) with the following thermocycling protocol: 10 min at 25 °C, 120 min at 37 °C, and a final step of 5 min at 85 °C.

RT-qPCR was performed using the QuantStudio™ 5 Real-Time PCR System. Each PCR reaction volume consisted of 4 µL of cDNA diluted to 1:10, 5 µL of SYBR™ Green Master Mix, and 0.5 µL of 10 µM forward and reverse primers of the reference/target gene (**Supplementary File 2**). The target genes included genes that are involved in inflammation (*interleukin 1β* [*il1β*]; *interleukin 8* [*il8*]; *tumour growth factor β* [*tgfb*]; *tumour necrosis factor* [*tnf*]), and oxidative stress responses (*glutathione-s-transferase* [*gsta*]; *glutathione peroxidase* [*gpx*], *manganese superoxide dismutase* [*mnsod*], and *catalase* [*cat*]). The thermocycling process was as follows: 2 min at 50 °C followed by pre-incubation at 95 °C for 20 s, then 40 cycles of 1 s at 95 °C and 30 s at 60 °C, and ending with a melt curve stage of 15 s at 95 °C, 1 min at 60 °C, and 15 s at 95 °C. To calculate amplification efficiencies, a 5-point standard curve of a 2-fold dilution series was prepared from pooled cDNA. The samples were run in duplicate and included minus reverse transcriptase and no-template controls. The relative gene expression was calculated by the 2^−ΔΔCt^ method using the geometric mean of *18 S ribosomal RNA* (*18s*, coefficient of variation = 4.96) and *elongation factor 1a* (*ef1a*, coefficient of variation = 2.41).

### Microbiota sequencing and bioinformatics

The dynamics of the microbial environment on the tank wall and the biomedia were determined through microbiota sequencing conducted by DNASense ApS (Aalborg, Denmark). Prior to DNA isolation, swabs were taken from all crevices of the 5 biomedia collected from an RAS unit. Briefly, DNA were isolated from the swabs using Molzym Ultra-Deep Microbiome kit (Molzym GmbH & Co. KG, Bremen, Germany). Amplicon libraries targeting variable regions 1–8 (bV18-A) of the bacterial 16 S rRNA gene were generated according to a custom protocol that was developed by the manufacturer.

Library preparation for Oxford Nanopore sequencing was performed using the SQK-LSK114 kit (Oxford Nanopore Technologies, UK) according to the manufacturer’s instructions, with modifications. Specifically, 500 ng of purified DNA were used as input, and all clean-up steps involved CleanNGS SPRI beads. The DNA concentration was quantified with the Qubit dsDNA HS Assay Kit (Thermo Fisher Scientific, USA). A subset of the amplicon libraries was assessed for size and purity using a TapeStation 2200 system and D1000/High Sensitivity D1000 ScreenTapes (Agilent, USA).

Sequencing was performed on a PromethION R10.4.1 flow cell using MinKNOW software (version 24.06.15). Basecalling and demultiplexing were conducted using MinKNOW Dorado version 7.4.14 with the high-accuracy configuration (r10.4.1_400bps_sup.cfg) and appropriate custom barcodes. After sequencing, reads were filtered for length (320–2000 bp) and quality (minimum Phred score > 17) using a local instance of filtlong v0.2.1 with the following parameters: --min_length 320 --max_length 2000 --min_mean_q 98. Taxonomic classification was performed using the Greengenes2 2022.10 reference database in RESCRIPt format obtained from QIIME. Before alignment, entries labelled as “uncultured,” “metagenome,” or “unassigned” were removed from the taxonomy file. The 16 S microbiome sequence data have been submitted to the NCBI’s Sequence Read Archive (SRA) as BioProject PRJNA1312769.

Read alignment to the Greengenes database was performed using minimap2 v2.24-r1122 with the -ax map-ont option, and alignment results were processed with samtools v1.14. Only alignments where the query sequence length deviated by less than 5% from the alignment length were retained. For quality control and denoising, reads were required to appear at least 10 times per sample. Operational taxonomic units (OTUs) representing less than 0.1% of the total mapped reads in each sample were excluded from further analysis. The count data and taxonomical data were analysed using the R packages phyloseq (version 1.52.0)^30^ and metacoder (version 0.3.8)^31^. A heatmap of the most abundant taxa was generated using the phyloseq implementation of NeatMap^32^.

### Statistics

Statistical analyses were performed using GraphPad Prism for Windows^®^ (version 8.0). Two-way analysis of variance (ANOVA) was used to evaluate differences in the changes within a treatment group over time and between groups at a specific time point. First, the data were checked for ANOVA assumptions, including tests for heteroscedasticity (Spearman’s test) and normality. Once these assumptions were met, Tukey’s multiple-comparison test was applied to identify significant differences between different comparisons. A significance level of *P* < 0.05 was used for all statistical tests. The histological results were analysed in a similar way using the R functions aov() and TukeyHSD() (from the base stats-package from R version 4.5.0) for two-way ANOVA and *post-hoc* tests.

## Results

### Susceptibility of *S. salmonicida* to LP and MP UV irradiation in situ

The cultures showed immediate signs of impact after LP UV treatment, with noticeable reductions in movement, particularly at doses exceeding 50 mJ/cm². An apparent dose‑related trend was observed with increasing UV doses, with higher doses generally associated with greater log reductions in the number of live parasites. The reductions were approximately twofold with each increase in dose (Fig. 1C). However, a 1-log reduction was only achieved at doses of 150 mJ/cm² and above. After 24 h, continued growth and active motility were observed by microscopy in cultures treated with 10 and 25 mJ/cm². Survival was slightly reduced at 50 mJ/cm², and no survival was observed at 75 mJ/cm² or higher (results not shown). After 96 h of exposure, slight movements were observed at doses of 25 mJ/cm², and the viability log reduction was no greater than 1.

Parasite cultures exposed to MP UV irradiation showed signs of impact starting at 10 mJ/cm², with markedly reduced or absent movement observed immediately after exposure, especially at doses above 25 mJ/cm² (Fig. 1D). A 5-log reduction in the number of live parasites was recorded immediately following exposure to doses of 50 mJ/cm² and above. In contrast, control samples exhibited vigorous motility, with parasites actively swarming and settling toward the bottom of the tubes. At 10 mJ/cm², limited movement was observed in a few individuals after 24 h. A 5-log reduction in viability was observed at 10 mJ/cm² after 48 h. Parasite motility ceased at doses > 10 mJ/cm² in 24 h, at which a log reduction of 5 was observed.

### Production performance and infection status following a simulated breach

During the trial, there were no inter-treatment differences in fish length (Fig. 2C), weight (Fig. 2D), or K-factor (Fig. 2E). Occasional scale loss accompanied by mild haemorrhaging was observed in some fish sampled at S3 (Fig. 2F). Still, no clear differences were identified between treatment groups. Additionally, sporadic liver discolouration characterised by light patches was noted in a few fish at S3 (Fig. 2G). Still, it could not be definitively linked to any specific treatment group.

Plasma, gills, head kidney, foregut, and hindgut samples from all fish were analysed for the presence of the parasite using RT-qPCR, which showed no positive results. A few fish showed detectable cycle threshold (CT) values, which remained above the defined threshold of 35 and were therefore not considered positive. Peritoneal fluid collected from selected fish at S3, which primarily exhibited liver discolouration, also tested negative in attempts to re-isolate the parasite. Similarly, swabs taken from the biomedia and tank walls returned negative results.

### Changes in key water quality parameters

**Fig. 3:**
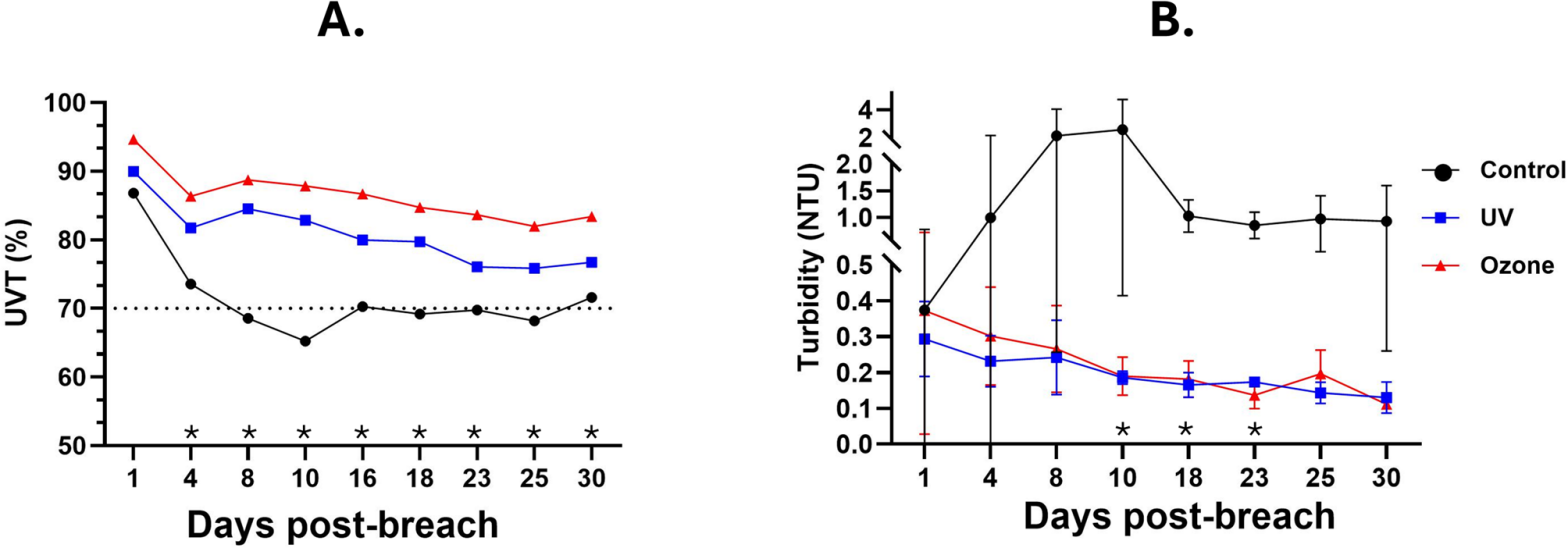
Water quality differences between the systems with and without RAS loop disinfection. Each experimental group had three modular RAS units. (**A**) Transmittance UVT (%) and (**B**) turbidity from 1 day after the breach until the end of the trial. These two parameters differed between the systems with RAS loop disinfection and the control. An asterisk (*) indicates that the control group differs significantly from the UV and ozone groups at the corresponding time point. All other parameters measured during the trial are provided in **Supplementary File 1**.

Higher values of UV transmittance (UVT, %) were observed in the groups that received RAS loop disinfection, and the ozone-treated group showed slightly higher values than the UV-treated group (Fig. 3A). In contrast, UVT in the control group fell below the threshold of 70% from day 8 post-breach. From days 4 to 30 post-breach, the control group consistently showed significantly lower UVT than the disinfected groups. Additionally, turbidity was significantly higher in the control group than in the groups that received RAS loop disinfection **(**Fig. 3B). Turbidity increased until day 10 post-breach, after which it remained stable for the rest of the trial. From days 10 to 23 post‑breach, turbidity was significantly higher in the control group than in the two treatment groups. All measured water quality parameters fluctuated over time during the trial, but no differences were observed between treatment groups (**Supplementary File 1**). All values remained within established thresholds that are considered optimal for this stage of salmon production.

### Quantitative morphological features of gills and skin

**Fig. 4:**
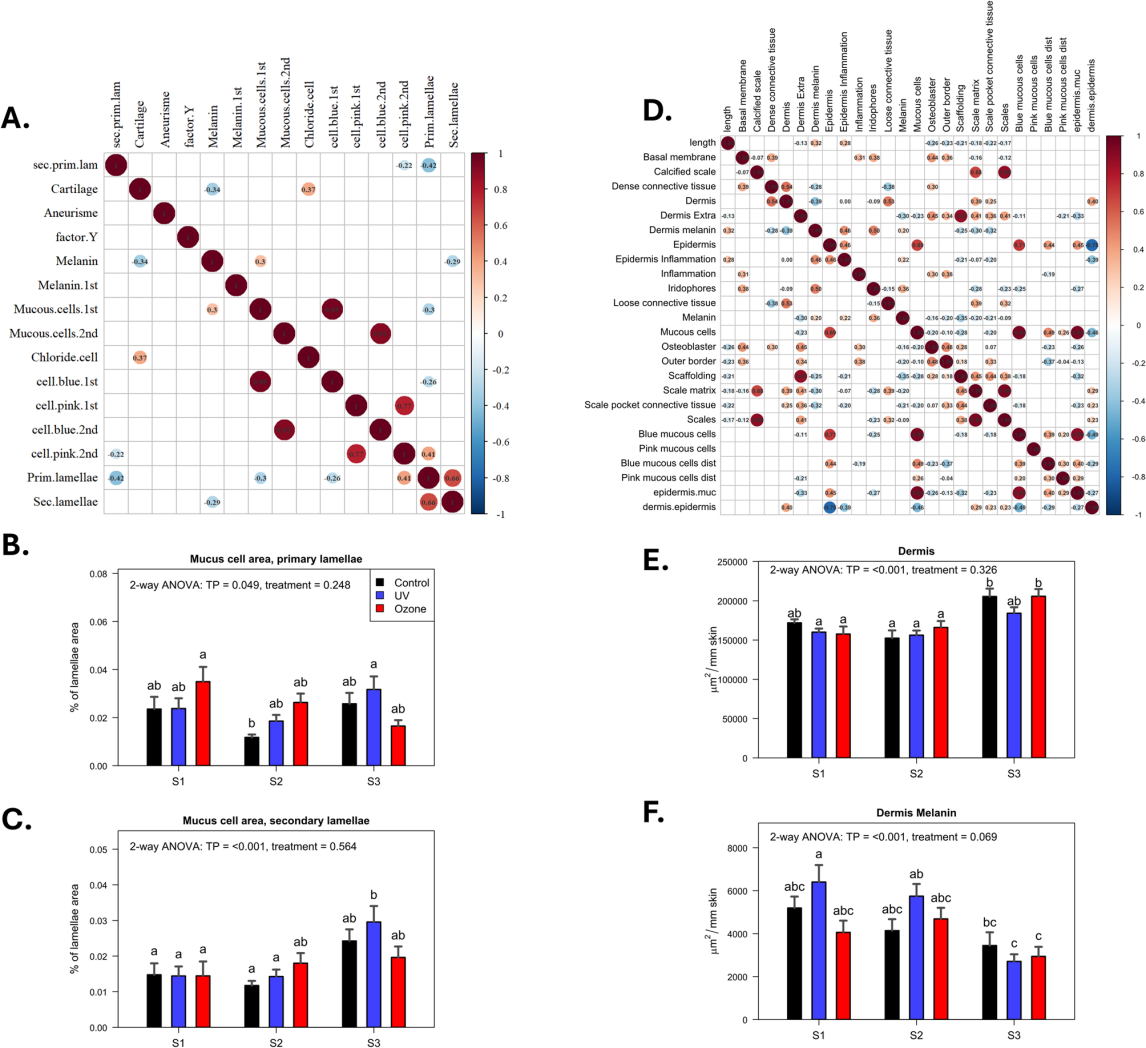
Histomorphology of mucosal organs. (**A**) Gills and (**D**) skin from fish sampled at all time points were analysed using an AI-based evaluation to assess key features of mucosal integrity. The correlation map of these features in each mucosal organ is colour-coded, with shades of blue indicating negative correlations and shades of red indicating positive ones. Pearson correlation coefficients are also shown on the map. Full results for all evaluated features are provided in **Supplementary Files 3–4**. Most parameters showed changes over time but not between treatment groups, including (**B–C**) mucous cell counts in the primary and secondary lamellae and (**E–F**) characteristics of the dermis and dermal melanin in the skin. Significant differences are indicated with different letters. *N* = 15 fish per experimental group at each time point.

Five parameters were analysed in the primary lamella (melanin area, chloride cell area, mucus cell area, blue mucus-cell number, pink mucus-cell number, and cartilage), while six parameters were assessed in the secondary lamella at three timepoints (melanin, granular cells, aneurism, mucus cell area, blue mucus cell number, pink mucus cell number, and cartilage) (Fig. 4A). Across all timepoints, there was a strong positive correlation between the mucus cell area and blue mucus cell number in the primary and secondary lamellae, as well as a strong correlation between pink mucus cell numbers in the two lamellar regions. However, none of the assessed parameters showed significant differences between treatment groups before (S1) or after the breach (S2–S3) (**Supplementary Files 3**).

Temporal changes were observed in several parameters, including the mucus cell area in the primary (Fig. 4B) and secondary lamellae (Fig. 4C) of the gills. For instance, in the control group, the mucus cell area in the secondary lamella was significantly higher at S3 than at S2. The melanin area was lowest at S3 across all treatment groups (**Supplementary File 3**). Additionally, while blue mucus cell numbers in the primary lamella remained stable over time, they peaked in the secondary lamella at S3, with significantly higher values occurring in the control group compared to S2 (**Supplementary File 3**).

A total of 26 parameters were included in the quantitative histological evaluation of the skin (Fig. 4D). There was a strong positive correlation between the epidermis area and both the mucus cell area and the number of blue mucus cells. A strong correlation was also found between the mucus-cell area and the number of blue mucus cells. Similar to the findings in the gills, most significant changes were associated with time rather than treatment effects (**Supplementary File 4**). For example, the dermis area in both RAS loop-disinfection groups was highest at S3 compared to the earlier timepoints (Fig. 4E). In contrast, dermal melanin levels decreased over time, with the control group showing a significantly lower level at S3 compared to S1 (Fig. 4F).

### Expression of inflammatory and oxidative stress response genes in mucosal organs

**Fig. 5:**
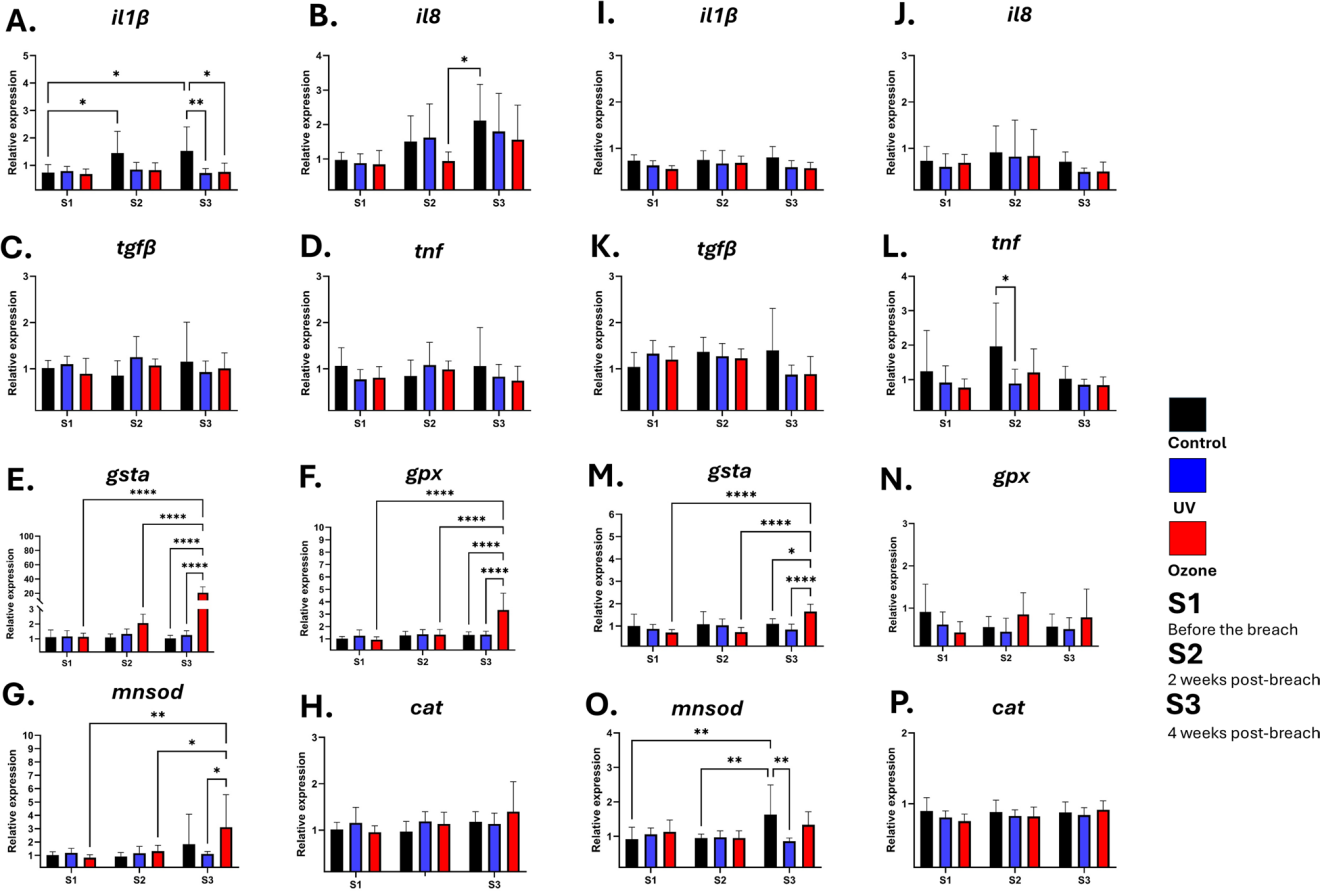
Gene expression profiles of the gills and skin. Expression levels of genes associated with inflammation (*il1β*,* il8*,* tgfβ*,* tnf*) and oxidative stress (*gsta*,* gpx*,* mnsod*,* cat*) were assessed in the (**A–H**) gills and (**I–P**) skin of fish sampled at all time points (S1–S3). Data are presented as mean + standard deviation (SD) based on 15 fish per experimental group at each time point. Statistically significant differences between the two groups are indicated by asterisks: *p <* 0.05 = *; *p <* 0.01 = **; *p <* 0.001 = ***; *p <* 0.0001 = ****. Note that the y-axis ranges differ across panels to accommodate variation in expression levels across the dataset. As the values are not absolute, axis scales were adjusted individually to optimise clarity and visualisation of the data.

Before the simulated breach (S1), no significant differences were observed among treatment groups in the expression of genes associated with inflammation and oxidative stress responses in the gills or skin (Fig. 5). In the gills, *il1β* expression in the control group significantly increased from S2 onward compared to S1 (Fig. 5A). By S3, *il1β* expression was lower in both RAS loop-disinfection groups relative to the control. For *il8*, expression in the gills of the ozone group at S2 was significantly different from that of the control group at S3 (Fig. 5B). No significant temporal or treatment-related changes were detected for *tgfβ* and *tnf* expression (Figs. 5C–D).

Ozone treatment notably affected genes related to oxidative stress in the gills (Figs. 5E–G). Expression levels of *gsta* (Fig. 5E), *gpx* (Fig. 5F), and *mnsod* (Fig. 5G) increased significantly over time, with peak expression occurring at S3. At this time point, *gsta* and *gpx* expression in the ozone group were significantly higher than in the control and UV groups (Fig. 5E–F). For *mnsod*, expression was elevated in the ozone group compared to the UV group at S3 but not significantly different from that of the control (Fig. 5G).

Changes in the expression of inflammatory response genes in the skin were generally limited (Fig. 5I–L). A significant difference was observed only for *tnf* expression, which was notably lower in the UV group compared to the control at S2 (Fig. 5L). Similar to *gsta* expression patterns in the gills, the expression in the skin of the ozone group exhibited an increasing trend over time, with significantly higher levels occurring at S3 than at earlier timepoints (Fig. 5M). Conversely, *mnsod* expression in the skin was significantly elevated in the control group at S3 compared to S1 and S2 (Fig. 5O). At S3, *mnsod* expression in the UV group was significantly lower than in the control.

**Fig. 6:**
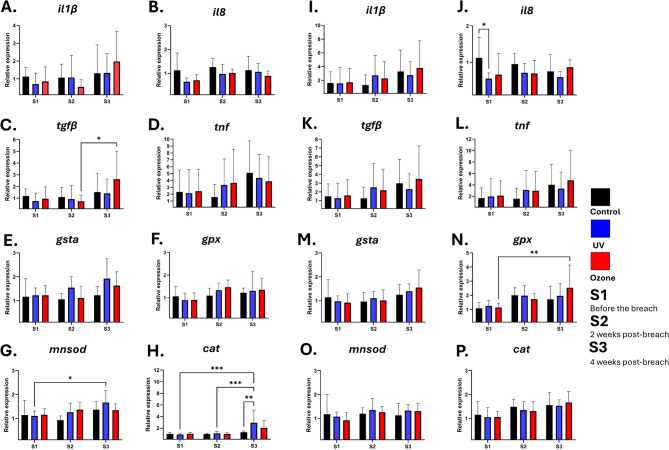
Gene expression profiles of the fore- and hindgut. Expression levels of genes associated with inflammation (*il1β*,* il8*,* tgfβ*,* tnf*) and oxidative stress (*gsta*,* gpx*,* mnsod*,* cat*) were assessed in the (**A–H**) foregut and (**I–P**) hindgut of fish sampled at all time points (S1–S3). Refer to Fig. 5 for additional information.

The expression of inflammatory and oxidative stress response genes in the foregut and hindgut was minimally influenced by time and treatment (Fig. 6). Inflammatory markers largely remained unchanged across sampling points and treatment groups, with the exception of *tgfβ* in the foregut (Fig. 6A–D, I–L). In the ozone group, *tgfβ* expression in the foregut at S3 was significantly elevated compared to S2 but not significantly different from S1 (Fig. 6C). Among oxidative stress genes, *mnsod* expression in the foregut of the UV group was significantly higher at S3 compared to S1, although not significantly different from S2 (Fig. 6G). The expression of *cat* in the foregut also showed a UV-induced response, with significantly higher levels occurring at S3 than at both earlier timepoints. Additionally, *cat* expression at S3 in the UV group was significantly higher than in the control. In the hindgut, gene expression of oxidative stress genes was largely unaffected by treatment except for *gpx*, which exhibited significantly higher expression at S3 than S1 (Fig. 6N).

### Microbiota of biomedia and tank wall

**Fig. 7:**
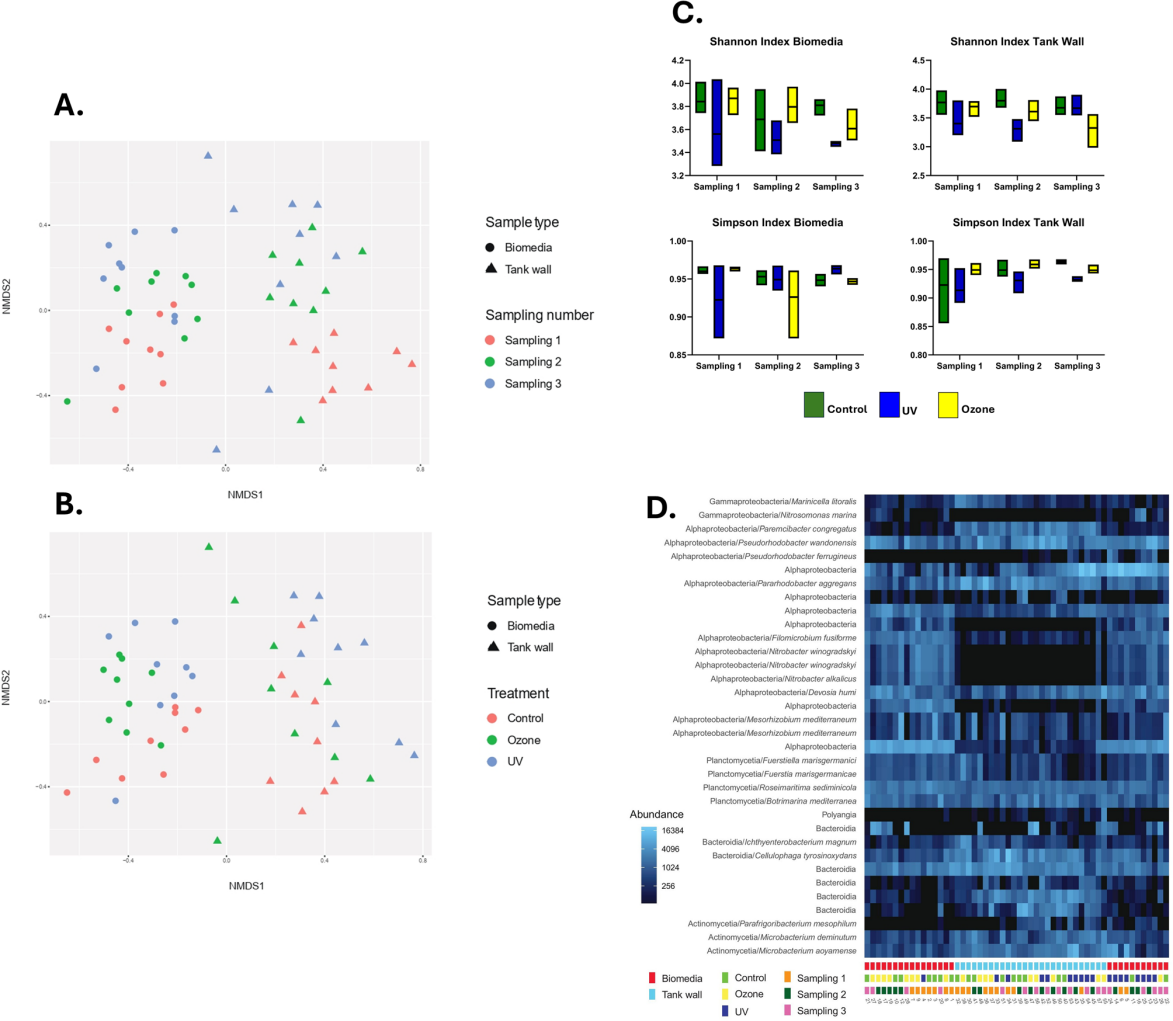
Microbial profiles of the tank wall and biomedia. (**A**) Non-metric multidimensional scaling (NMDS) was used to compare various factors, such as sample type, in relation to (**A**) sampling time points and (**B**) treatment groups. (**C**) Shannon and Simpson alpha diversity indices were calculated to assess microbial diversity in the biomedia and tank wall over time and between treatment groups. (**D**) Heatmap of OTUs (class/species (where available)) that represented at least 5% of reads in at least one sample. Sample types, treatment groups, and sampling time points are colour-coded to illustrate relationships among the different factors.

Microbiota analysis yielded an average of 122,933 raw reads and 61,246 mapped reads per sample, with a mean of 94 OTUs identified (**Supplementary File 5**). NMDS analysis showed distinct clustering by sample type, with biomedia samples grouping on the left and tank wall samples on the right of the plot (Fig. 7A). Sampling time had little effect on biomedia microbial profiles, whereas tank wall samples from sampling 1 were separated from later time points. While a slight difference was observed between the control and ozone treatments in biomedia, the microbial profiles of the control and UV‑treated groups showed partial overlap, with no strong separation (Fig. 7B). No treatment‑related separation was observed in tank wall samples.

Shannon and Simpson diversity indices were stable over time and did not differ between treatments or sampling locations (Fig. 7C). Mean diversity was similar between biomedia and tank wall samples across all treatments (Shannon: biomedia = 3.68, tank wall = 3.58; Simpson: biomedia = 0.95, tank wall = 0.94). Greater variability was observed in the UV group at sampling 1, but this decreased at later time points.

The most abundant taxa (Fig. 7D) were dominated by Gammaproteobacteria, Alphaproteobacteria, Actinomycetia, and Bacteroidia. Biomedia samples clustered into two groups: one dominated by the control and ozone treatments, with some separation by sampling time, and enriched in Alphaproteobacteria, and a second largely composed of UV‑treated samples with little influence of time. In contrast, tank wall samples formed a single cluster with no clear grouping by treatment or sampling time among dominant taxa.

**Fig. 8:**
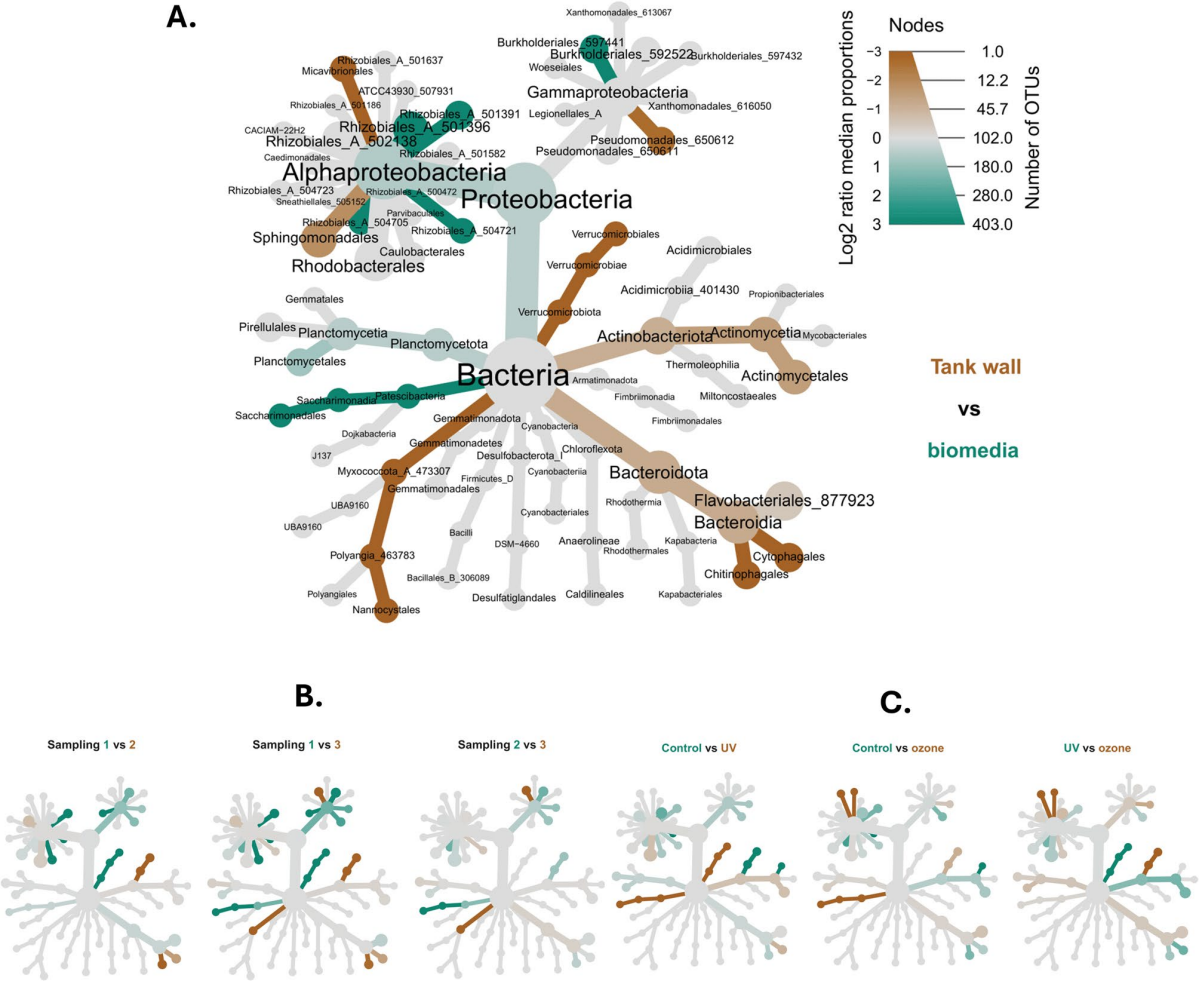
Metacoder heat-tree overview of the microbiota composition. (**A**) Heat tree illustrating the differences between the tank wall and biomedia. Colours reflect the log₂ ratio of median read proportions between sampling locations. Taxa enriched in the biomedia are shown in green, while those enriched on the tank wall are shown in brown. Heat trees are further separated according to (**B**) sampling time points and (**C**) treatment groups. Enrichment colour coding and taxonomy are shown in **A**.

The Metacoder heat‑tree visualisation summarised the overall microbial composition and revealed differences driven mainly by sampling location, time, and treatment (Fig. 8). Proteobacteria were dominated by Alphaproteobacteria and Gammaproteobacteria (Fig. 8A). Tank wall samples were enriched in Verrucomicrobiota, Actinobacteriota, Bacteroidota, and Myxococota, whereas biomedia samples showed higher abundances of Patescibacteria, Saccharimonadales and Saccharimonadia. Within Alphaproteobacteria, Rhizobiales was enriched in the biomedia, while Sphingomonadales and Micavibrionales were enriched on the tank wall. Similarly, within Gammaproteobacteria, Pseudomonadales was enriched on the tank wall, whereas Burkholderiales was enriched in the biomedia.

To further examine these patterns, microbial differences were assessed across sampling time points and treatments (Figs. 8B–C). Comparisons across time points showed distinct heat‑tree profiles, with consistent enrichment of several microbial groups, particularly when sampling 1 was compared with samplings 2 and 3. For example, Verrucomicrobiota was enriched at sampling 1, while Acidimicrobiia and Acidimicrobiales were more abundant at sampling 2, consistent with the patterns observed in both comparisons: sampling 1 versus 2 and sampling 1 versus 3. In the comparison of sampling 1 versus 3, Patescibacteria, Saccharimonadia, Saccharimonadales, Verrucomicrobiota, and several representatives from Gammaproteobacteria were enriched in sampling 1, whereas Myxococcota was more abundant in sampling 2.

Comparisons among treatments revealed distinct microbial signatures. In the comparison between the control and UV treatment, Verrucomicrobiota, Verrucomicrobiae, Verrucomicrobiales, Patescibacteria, Saccharimonadia, and Saccharimonadales were enriched in the UV group, whereas Acidimicrobiia and Acidimicrobiales were more abundant in the control group. The comparisons between the control versus ozone treatment, as well as the UV treatment versus ozone treatment, showed some overlap, with the ozone group exhibiting enrichment in Micavibrionales, Rhizobiales, Acidimicrobiia, and Acidimicrobiales. In the comparison of UV versus ozone treatments, the UV group showed higher abundances of Verrucomicrobiota, Verrucomicrobiae, Verrucomicrobiales, Actinobacteriota, Actinomycetia, and Actinomycetales than the ozone group.

## Discussion

Infectious diseases continue to pose a persistent biological challenge in Atlantic salmon aquaculture, despite the recent adoption of new production systems and technologies aimed at improving biosecurity. Unlike other parasitic infections, such as sea lice and amoebic gill disease, which are recorded annually, spironucleosis caused by the diplomonad *S. salmonicida* occurs infrequently, sometimes with intervals as long as 10 years in Northern Norway. Although outbreaks are sporadic, their impact can be severe and highly damaging. This highlights the importance of preventive biosecurity measures and particularly effective disinfection. This study found that *S. salmonicida* is sensitive to UV treatment in situ. However, a simulated biosecurity breach in a RAS revealed complex interactions among the parasite, disinfection methods, and the host salmon.

### Parasite sensitivity to UV treatment in culture

UV treatment is an established approach for pathogen inactivation in aquaculture^13,14,21^. However, previously defined threshold doses for various pathogens may need to be reassessed as developments continue to occur in both UV technologies (e.g., the shift from LP to MP lamps) and aquaculture systems (e.g., flow-through to RAS; intake water versus RAS loop disinfection). We demonstrated that *S. salmonicida* is susceptible to both LP and MP UV irradiation in situ, with MP UV showing greater efficacy at lower doses.

Under LP UV treatment, a clear dose-dependent reduction in parasite viability was observed, but relatively high doses were required to achieve effective inactivation. A 1-log reduction was only achieved at 150 mJ/cm², and parasites exposed to lower doses (10–25 mJ/cm²) showed limited reduction in viability and likely recovered as growth was still observed after 96 h post-exposure. These findings indicate that *S. salmonicida* exhibits a certain level of resistance to LP UV, which limits its practical effectiveness unless higher doses are applied. This result partly reflects findings in *G. intestinalis*, a closely related parasite to *S. salmonicida*, which was shown to tolerate LP UV exposure doses of up to 100 mJ/cm² ^19^.

In contrast, MP UV treatments induced a more rapid and pronounced effect with a 5-log reduction corresponding to 99.999% effectiveness observed at 50 mJ/cm² and above. Notably, even the lowest MP doses (10–25 mJ/cm²) resulted in significant reductions in viability. The better inactivation achieved with MP UV likely reflects the broader wavelength range and stronger photochemical damage that it causes compared to LP UV^18,33^. Despite these promising results, it is important to note that the tests were conducted using a high concentration of parasites in water with low salinity and minimal organic load. In practical settings such as hatcheries, several factors can significantly influence the effectiveness of UV treatment, such as the level of organic matter and particle characteristics in the water^34^. Therefore, these conditions must be carefully considered when determining the appropriate UV dose under farming conditions.

### Simulated biosecurity breach

Although not identified unequivocally, one of the transmission routes being considered for the parasite involves the intake water during the freshwater stage of production, particularly in Northern Norway, where outbreaks have been recorded in recent years^26,35^. These indications formed the basis for simulating a biosecurity breach via the water intake in the present study, which resulted in no evidence of *S. salmonicida* infection in any of the sampled fish, regardless of tissue type or treatments (with or without RAS loop disinfection). Occasional gross pathologies, such as mild haemorrhaging and scale loss, were observed in a few fish at S3, but these were not consistently associated with any specific treatment group. Attempts to re-isolate the parasite from the peritoneal fluid of fish showing visible unspecific pathologies were unsuccessful, which further suggests that an active infection was likely not established. These findings suggest several possibilities: (1) transmission via intake water may not be an effective route for inducing disease in salmon reared in RAS; (2) the experimental biosecurity breach model employed in this study may not have been sufficiently effective at establishing infection, or (3) the dose and the frequency of breach may not be optimal. Nonetheless, our results challenge anecdotal assumptions and suggest that the transmission and infection dynamics may be more complex than previously believed. It is also possible that the parasite used in the trial had reduced infectivity or pathogenicity at the time of exposure. Importantly, the parasites were derived from clonal cultures originating from a recent isolate obtained from salmon diagnosed with spironucleosis, suggesting that loss of virulence is unlikely but cannot be entirely excluded. Future studies should also examine whether host stress plays a critical role in modulating susceptibility to *S. salmonicida*. Although mucosal oxidative stress responses were documented in the present study, these effects may not have been sufficiently pronounced to predispose the fish to successful parasite establishment and disease development.

We could not conclude that the RAS loop disinfection with UV or ozone may have effectively prevented parasite transmission since there were no clinical signs of the disease or the parasite in the fish from the control group as well. We did not monitor the viability of the parasite immediately after its introduction into the RAS units, so it remains unclear whether the parasite died shortly after being added. However, laboratory-scale microbiological trials have shown that at salinities between 10 and 15 ppt, the parasite can survive for 5 to 10 h^36^. Moreover, we found no evidence that the parasite established itself in the system, which helps explain the absence of clinical signs of infection. While our tracing in the system was not exhaustive, the absence of the parasite in two key locations, the tank walls and biomedia, strongly suggests that *S. salmonicida* could not become established in the experimental RAS units, regardless of whether RAS loop disinfection was applied. Future research should focus on identifying other potential vectors of the parasite in RAS, such as infected fish, sludge or contaminated biomedia, as well as investigating which RAS conditions might support the proliferation and establishment of the parasite following a biosecurity breach.

Although the simulated breach did not lead to infection, the effects of the breach and the RAS loop disinfection protocol can still be evaluated in terms of their impact on fish health and welfare. Key growth metrics remained comparable across treatments throughout the study, including fish length, weight, and condition factor (K-factor). This suggests that implementing UV or ozone RAS loop disinfection did not negatively impact fish growth or health under the conditions tested. Similar findings have been reported in previous studies, where UV and ozone treatments in RAS systems did not compromise fish performance metrics when properly managed^37^.

Water quality dynamics further support the impact of disinfection on system conditions. UV transmittance was consistently higher in the UV and ozone groups, which indicated improved water clarity and lower levels of dissolved organic matter. The ozone group showed slightly better UVT values than the UV group, which suggests a potentially greater effect of ozone on turbidity under these conditions, as observed previously^23^. In contrast, UVT in the control group declined significantly after the breach and fell below the recommended 70% threshold^38^.

Turbidity trends mirrored UVT, with the control group exhibiting significantly higher turbidity levels, particularly in the early days post-breach. This suggests that the absence of RAS loop disinfection allowed for greater organic accumulation. While water quality parameters fluctuated over time, as expected in dynamic RAS environments, all remained in acceptable limits for optimal fish production^39–41^. These findings highlight the potential benefits of continuous disinfection in maintaining water quality following a biosecurity breach without compromising welfare or performance.

### Mucosal integrity and mucosal oxidative stress response

The fish neither exhibited clinical signs of spironucleosis nor tested positive for the parasite, so the health and welfare assessments focused on evaluating the effects of the RAS environment and loop disinfection on fish, particularly on mucosal health. Previous research has shown that various conditions in RAS can affect the gills and skin, including water quality parameters, disinfection practices, and other environmental stressors^23,41,42^. For example, elevated ozone levels have been shown to cause necrosis and other gill pathologies, which compromise both barrier and respiratory functions^24^. Nevertheless, when maintained at optimal levels, ozone can help create a rearing environment that supports good gill health^23^.

Unlike ozone, the effects of UV-treated water on fish, including Atlantic salmon, are not well documented^41^. RAS loop disinfection, particularly UV treatment, had a minimal impact on the mucosal integrity of the gills and skin of Atlantic salmon in the present study. Most observed changes were time-dependent rather than treatment-driven, which suggests that these changes are part of the natural developmental trajectory rather than a response to the RAS loop disinfection or, to a lesser extent, the simulated breach.

However, molecular analyses provided evidence that ozone treatment triggered an oxidative stress response, particularly in the gills and skin. Expression of oxidative stress-related genes such as *gsta*, *gpx*, and *mnsod* significantly increased over time in the ozone group, and the highest levels were observed at the final sampling point. This response aligns with the established effects of ozone exposure on the oxidative stress axis, where prolonged exposure can lead to the production of reactive oxygen species and activate the antioxidant defence mechanisms of an organism. In both salmon and juvenile turbot (*Psetta maxima*), exposure to ozone has been shown to influence the expression of various genes involved in antioxidant defence, which particularly suggests the activation of protective mechanisms in response to oxidative stress triggered by ozone^23,24,43^. At present, we cannot conclude whether the elevated expression of oxidative stress markers has negative health implications. However, histological assessments suggest that the activation of mucosal oxidative stress did not lead to overt tissue damage or compromised organ health.

The expression of *il1β*, a key regulator of inflammation, showed distinct patterns in the gills. In the control group, *il1β* expression increased over time, which may reflect a gradual upregulation of inflammatory processes that may be potentially linked to rising turbidity levels observed in the system. In contrast, the expression of pro-inflammatory genes such as *il1β* and *tnf* was lower in the ozone and UV groups compared to the control at termination. It remains unclear whether the rearing environment created by loop disinfection directly contributes to the lower gene expression of these pro-inflammatory markers, given that UVT and turbidity were the only water quality parameters differing between treatments. Lastly, the two gut regions showed limited responsiveness to the treatments as the significant pairwise comparisons did not present a clear or consistent pattern. The gut plays an important role in the pathogenesis of spironucleosis^9^, so the observed gene expression profile supports the likelihood that infection did not occur.

### Influence on microbial communities in biomedia and tank wall

The microbial community profiling revealed sample-specific, timepoint-driven, and to some extent treatment-associated patterns, which highlight the complex microbial dynamics in RAS. As documented in other RAS studies^44,45^, the biomedia and tank walls supported distinct microbial communities, with the NMDS plot indicating consistent clustering between the two sample types. The biomedia supported more stable microbial communities with minimal temporal fluctuations, which was likely due to its high surface area design and promotion of persistent biofilm formation. In contrast, the microbial assemblages on the tank walls exhibited greater temporal variability, particularly between the first and subsequent sampling points, although this variability could not be attributed to the biosecurity breach.

These differences between the two sampling locations may be attributed to the differences in flow dynamics, nutrient exposure, and surface properties, which influence the biofilm formation of these microbial communities^44,46^. The Shannon and Simpson alpha diversity indices were comparable between sampling locations, which contrasted with observations in commercial production^44^. The discrepancies in observations may stem from the differing production intensities and primary microbial succession processes between small-scale experimental units and commercial-scale systems.

Taxonomic profiling revealed several key bacterial groups predominating in the system, including Alphaproteobacteria, Gammaproteobacteria, Actinomycetia, and Bacteroidia, which are commonly reported taxa in RAS biofilms^44,47,48^. Significant temporal variations in the microbial composition of the biomedia suggest that the system underwent continuous maturation throughout the study period. Moreover, disinfection protocols had a greater impact on the microbial composition of the tank walls than on the biomedia, which was likely due to differences in material properties and quality. These factors influence microbial colonisation and biofilm development, which have been reported to contribute to biofilm formation^49^.

UV treatment was associated with enrichment of Verrucomicrobiota and Saccharimonadales, whereas ozone treatment favoured the presence of Rhizobiales and Acidimicrobiales. These shifts in community structure likely reflect the selective pressures imposed by different disinfection strategies, which can suppress sensitive taxa while promoting the proliferation of more resistant groups. A similar pattern has been observed previously with ozone disinfection, where microbial dynamics were marked by notable changes in the dominant taxa in the community^50,51^. The impact of UV treatment on microbial dynamics depends significantly on whether it is applied upstream or downstream in the system^45^. Despite these changes, the consequences at the system level were minimal, as water quality parameters remained the same in all treatment groups during the trial, particularly for the nitrogen compounds.

## Conclusions

Effective biosecurity, which limits the entry and proliferation of *S. salmonicida*, has been the only viable preventive strategy against this enigmatic pathogen. In this study, we demonstrated that *S. salmonicida* is susceptible to UV treatment under in situ conditions. MP UV showed better inactivation efficiency than LP UV, and complete inactivation with no signs of regrowth occurred at doses of 50 mJ/cm² and above. Future studies should examine the influence of other water quality parameters on the effectiveness of UV disinfection, such as organic compounds. The simulated parasite entry via intake water in the RAS did not result in infection or detection of the parasite in the system, but it remains unclear whether this outcome was due to the low efficiency of waterborne transmission or to limitations in the experimental infection model. RAS loop disinfection using UV or ozone provides a good tank environment with low turbidity and has no adverse effects on welfare, performance, or mucosal health. However, gene expression profiling in key mucosal organs revealed that ozone likely triggered oxidative stress in the gills, suggesting that its application must be carefully managed, despite its beneficial effects on water quality.

Microbial community profiling revealed distinct assemblages between biomedia and tank wall surfaces, which underscores the importance of sampling location when monitoring RAS microbiotas. Furthermore, RAS disinfection influenced the microbial community structure by selectively promoting or suppressing specific taxa, and these disinfection-induced microbial changes had a significant impact at the system level. Together, these findings support the potential of UV treatment as a promising disinfection measure against *S. salmonicida* and emphasise the need to account for the complex interactions between the fish, pathogen, and rearing environment when designing RAS disinfection as part of practical biosecurity strategies.

## Supplementary Information

Below is the link to the electronic supplementary material.

Supplementary Material 1

Supplementary Material 2

Supplementary Material 3

Supplementary Material 4

Supplementary Material 5

## Data Availability

The 16 S microbiome sequence data reported in this paper has been deposited to the NCBI’s Sequence Read Archive (SRA) as BioProject PRJNA1312769.
