# Supplementary material for: Interplay between disinfection and the enigmatic diplomonad parasite Spironucleus salmonicida in Atlantic salmon
Source: Sci Rep. 2026 May 8;16:21163. doi: 10.1038/s41598-026-51626-4 (PMC13341783; doi:10.1038/s41598-026-51626-4)
Supplement: Supplementary file 2 — Supplementary Material 2 [file 41598_2026_51626_MOESM2_ESM.docx]

**Supplementary File 2: Primers used in the study.**

| Category | Gene name | Abbreviation | Sequences (5’ 🡪 3’) | Reference | Efficiency |
| --- | --- | --- | --- | --- | --- |
| Reference genes | *18s ribosomal RNA* | *18s* | F: GCCCTATCAACTTTCGATGGTAC  R: TTTGGATGTGGTAGCCGTTTCTC | (Skugor et al., 2009) | 94.62 |
|  | *Elongation factor 1a* | *elfa1* | F: GAATCGGCTATGCCTGGTGAC  R: GAATCGGCTATGCCTGGTGAC | (Furtado et al., 2022) | 99.05 |
| Parasite | *S. salmonicida* | *18s_Spiro* | F: GCGAGATTTGTTGCATTTAAAAAG  R: GCACATGCAGTGTCCAATCG  Probe: ACACGGAGAGTATTCT | (Miller et al., 2016) | NA |
| Inflammation | *Interleukin 1b* | *il1β* | F: GTATCCCATCACCCCATCAC  R: TTGAGCAGGTCCTTGTCCTT | (Emam et al., 2022) | 96.90 |
|  | *Interleukin 8* | *il8* | F: GAAAGCAGACGAATTGGTAGAC  R: GCTGTTGCTCAGAGTTGCAAT | (Zanuzzo et al., 2020) | 95.94 |
|  | *Tumor necrosis factor alpha* | *tnf* | F: GTGTATGTGGGAGCAGTGTT  R: GAAGCCTGTTCTCTGTGACT | (Marcos-López et al., 2018) | 92.99 |
|  | *Transforming growth factor beta* | *tgfβ* | F: GCCATCCGTGGACAGATACT  R: TCTCCCTCCTGGTCAATCTCT | (Marcos-López et al., 2018) | 94.38 |
| Oxidative stress | *Catalase* | *cat* | F: GGGCAACTGGGACCTTACTG  R: GCATGGCGTCCCTGATAAA | (Olsvik et al., 2011) | 101.80 |
|  | *Glutathione S-transferase* | *gsta* | F: AGGGCACAAGTCTAAAGAAGTC  R: GTCTCCGTGTTTGAAAGCAG | (Lazado and Voldvik, 2020) | 100.30 |
|  | *Glutathione peroxidase* | *gpx* | F: GATTCGTTCCAAACTTCCTGCTA  R: GCTCCCAGAACAGCCTGTTG | (Solberg et al., 2012) | 98.20 |
|  | *Manganese superoxide dismutase* | *mnsod* | F: GTTTCTCTCCAGCCTGCTCTAAG  R: CCGCTCTCCTTGTCGAAGC | (Solberg et al., 2012) | 96.70 |

*NA = Not applicable*

Emam, M., Caballero-Solares, A., Xue, X., Umasuthan, N., Milligan, B., Taylor, R.G., Balder, R., Rise, M.L., 2022. Gill and Liver Transcript Expression Changes Associated With Gill Damage in Atlantic Salmon (Salmo salar). Frontiers in Immunology 13. <https://doi.org/10.3389/fimmu.2022.806484>

Furtado, F., Breiland, M.W., Strand, D., Timmerhaus, G., Carletto, D., Pedersen, L.-F., Afonso, F., Lazado, C.C., 2022. Regulation of the molecular repertoires of oxidative stress response in the gills and olfactory organ of Atlantic salmon following infection and treatment of the parasite Neoparameoba perurans. Fish & Shellfish Immunology 130, 612–623. <https://doi.org/https://doi.org/10.1016/j.fsi.2022.09.040>

Lazado, C.C., Voldvik, V., 2020. Temporal control of responses to chemically induced oxidative stress in the gill mucosa of Atlantic salmon (Salmo salar). J Photochem Photobiol B 205, 111851. <https://doi.org/10.1016/j.jphotobiol.2020.111851>

Marcos-López, Calduch-Giner, J.A., Mirimin, L., MacCarthy, E., Rodger, H.D., O’Connor, I., Sitjà-Bobadilla, A., Pérez-Sánchez, J., Piazzon, M.C., 2018. Gene expression analysis of Atlantic salmon gills reveals mucin 5 and interleukin 4/13 as key molecules during amoebic gill disease. Scientific Reports 8(1), 13689. <https://doi.org/10.1038/s41598-018-32019-8>

Miller, K.M., Gardner, I.A., Vogerstichel, R., Burnley, T., Schulze, A.D., Li, S., Tabata, A., Kaukinen, K.H., Ming, T.J., Ginther, N.G., 2016. Report on the performance evaluation of the Fluidigm BioMark platform for high-throughput microbe monitoring in salmon.

Olsvik, P.A., Torstensen, B.E., Hemre, G.-I., Sanden, M., Waagbø, R., 2011. Hepatic oxidative stress in Atlantic salmon (Salmo salar L.) transferred from a diet based on marine feed ingredients to a diet based on plant ingredients. Aquaculture Nutrition 17(2), e424–e436. <https://doi.org/https://doi.org/10.1111/j.1365-2095.2010.00778.x>

Skugor, S., Jørgensen, S.M., Gjerde, B., Krasnov, A., 2009. Hepatic gene expression profiling reveals protective responses in Atlantic salmon vaccinated against furunculosis. BMC Genomics 10, 503. <https://doi.org/10.1186/1471-2164-10-503>

Solberg, M.F., Kvamme, B.O., Nilsen, F., Glover, K.A., 2012. Effects of environmental stress on mRNA expression levels of seven genes related to oxidative stress and growth in Atlantic salmon Salmo salar L. of farmed, hybrid and wild origin. BMC Research Notes 5(1), 672. <https://doi.org/10.1186/1756-0500-5-672>

Zanuzzo, F.S., Beemelmanns, A., Hall, J.R., Rise, M.L., Gamperl, A.K., 2020. The innate immune response of Atlantic salmon (*Salmo salar*) is not negatively affected by high temperature and moderate hypoxia. Frontiers in Immunology Volume 11 - 2020. <https://doi.org/10.3389/fimmu.2020.01009>
