# Supplementary material for: Interplay between disinfection and the enigmatic diplomonad parasite Spironucleus salmonicida in Atlantic salmon
Source: Sci Rep. 2026 May 8;16:21163. doi: 10.1038/s41598-026-51626-4 (PMC13341783; doi:10.1038/s41598-026-51626-4)
Supplement: Supplementary file 3 — Supplementary Material 3 [file 41598_2026_51626_MOESM3_ESM.pdf]

Supplementary File 1

Additional water quality parameters and variables monitored during the trial

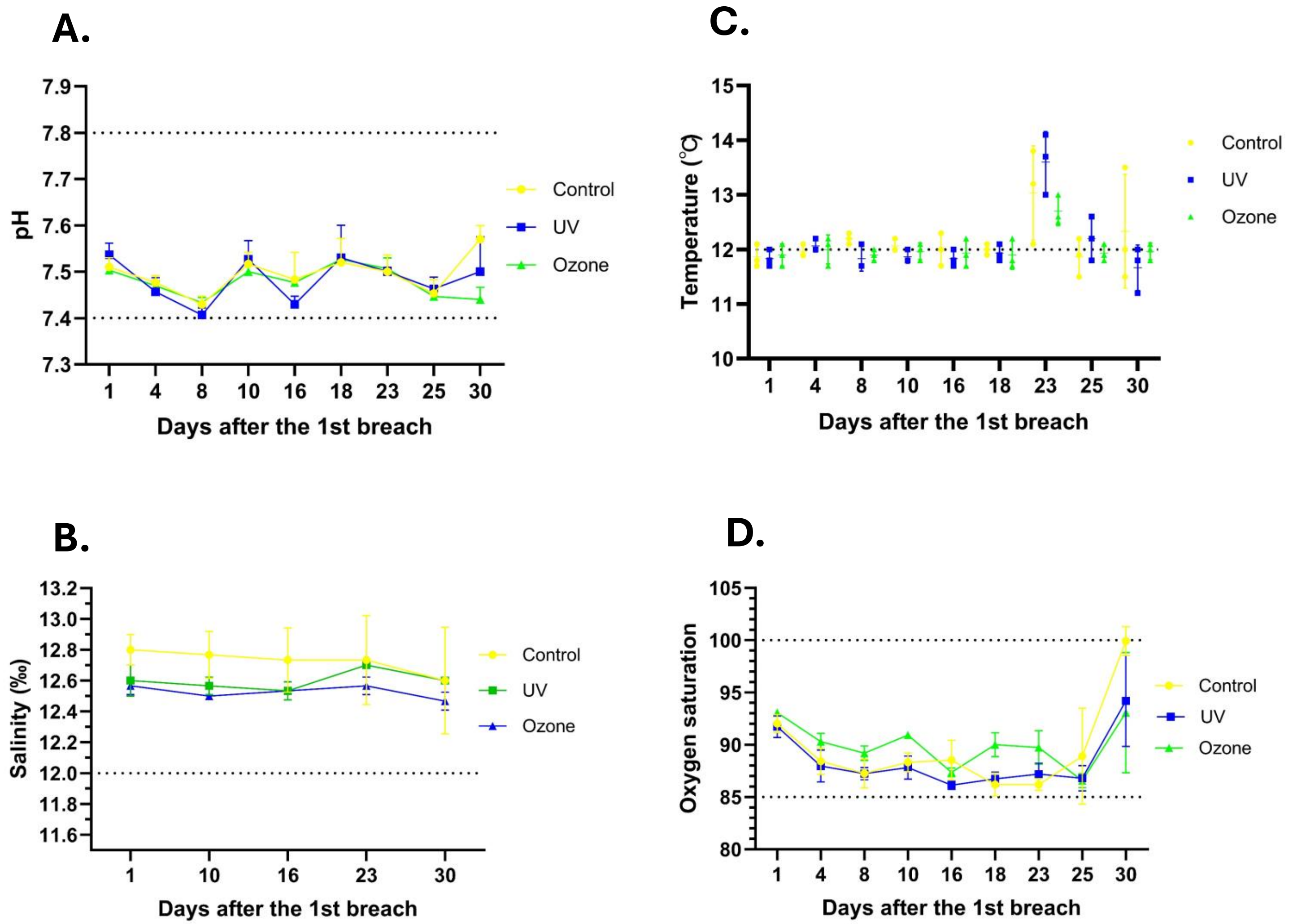

Supplementary File 2A. Additional water quality parameters monitored during the trial. Temporal changes in (A) pH, (B) salinity, (C) temperature, and (D) oxygen saturation following the breach. The dotted line in panels C and D indicates the average target value, while the two dotted lines in panels A and D represent the upper and lower threshold limits. Each treatment group had 3 replicate RAS units.

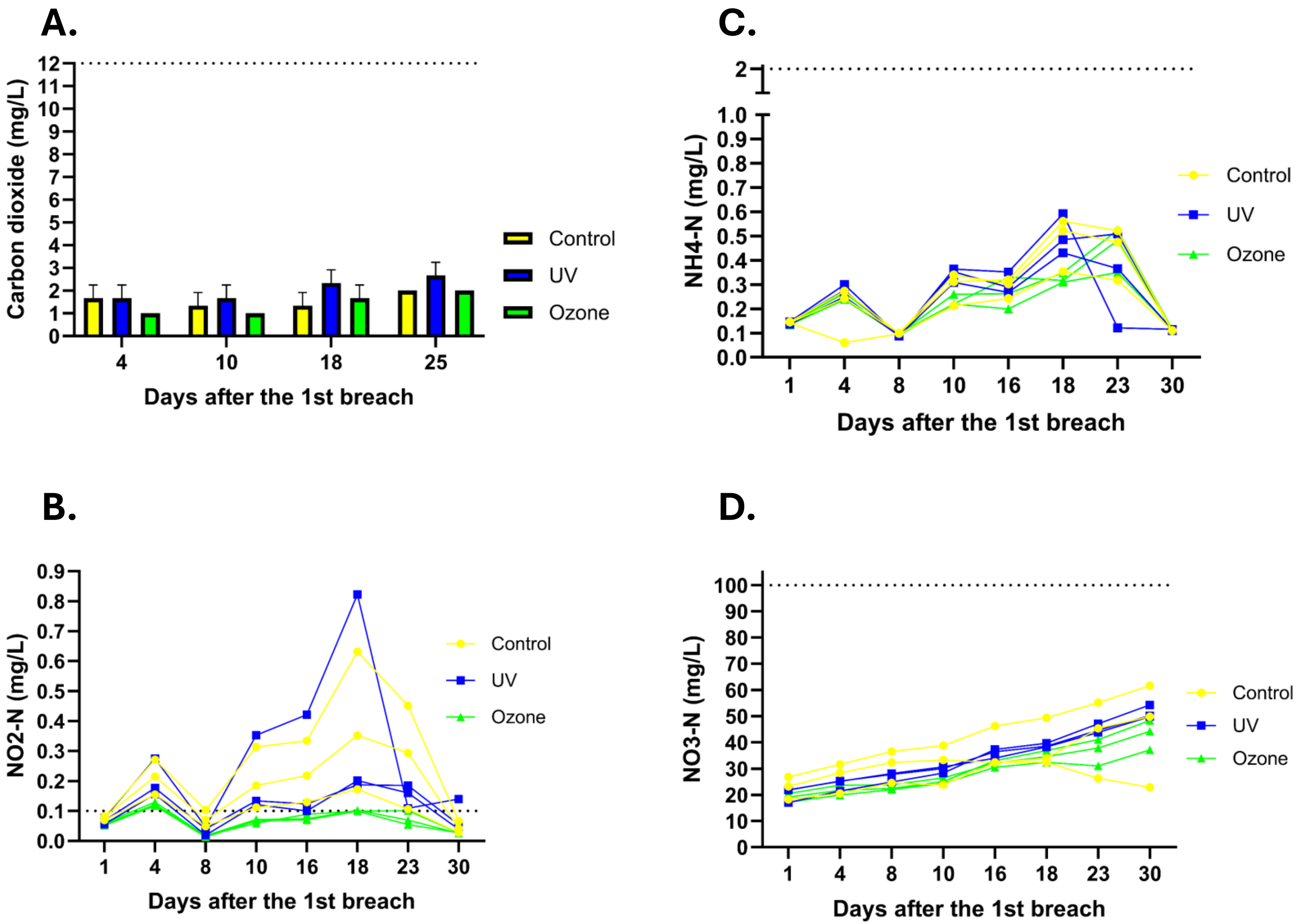

Supplementary File 2B. Additional water quality parameters monitored during the trial. Temporal changes in (A) CO_2, (B) TAN, (C) nitrite, and (D) nitrate following the breach. The dotted line indicates threshold limits. Note that for C-D, the values per replicate RAS unit are provided. Each treatment group had 3 replicate RAS units.
