# Supplementary material for: Interplay between disinfection and the enigmatic diplomonad parasite Spironucleus salmonicida in Atlantic salmon
Source: Sci Rep. 2026 May 8;16:21163. doi: 10.1038/s41598-026-51626-4 (PMC13341783; doi:10.1038/s41598-026-51626-4)
Supplement: Supplementary file 4 — Supplementary Material 4 [file 41598_2026_51626_MOESM4_ESM.pdf]

Supplementary File 3

Complete list of parameters histomorphometrically assessed in the gills

A region of interest (ROI) was defined for each sample (one ROI per sample) and analysed using the Aiforia® AI Gill model, which was trained on at least 3,370 annotated regions derived from 97 samples across eight projects. Variables in the gill dataset were normalised to the total lamellar area of each sample and are presented as a percentage of that area. Values shown represent data from 15 individual fish per sampling point in each treatment group. Statistical significance in each parameter was assessed using two-way ANOVA to evaluate changes within groups over time and differences between groups at specific time points. Significant differences are indicated by different letter annotations, and corresponding p-values are provided.

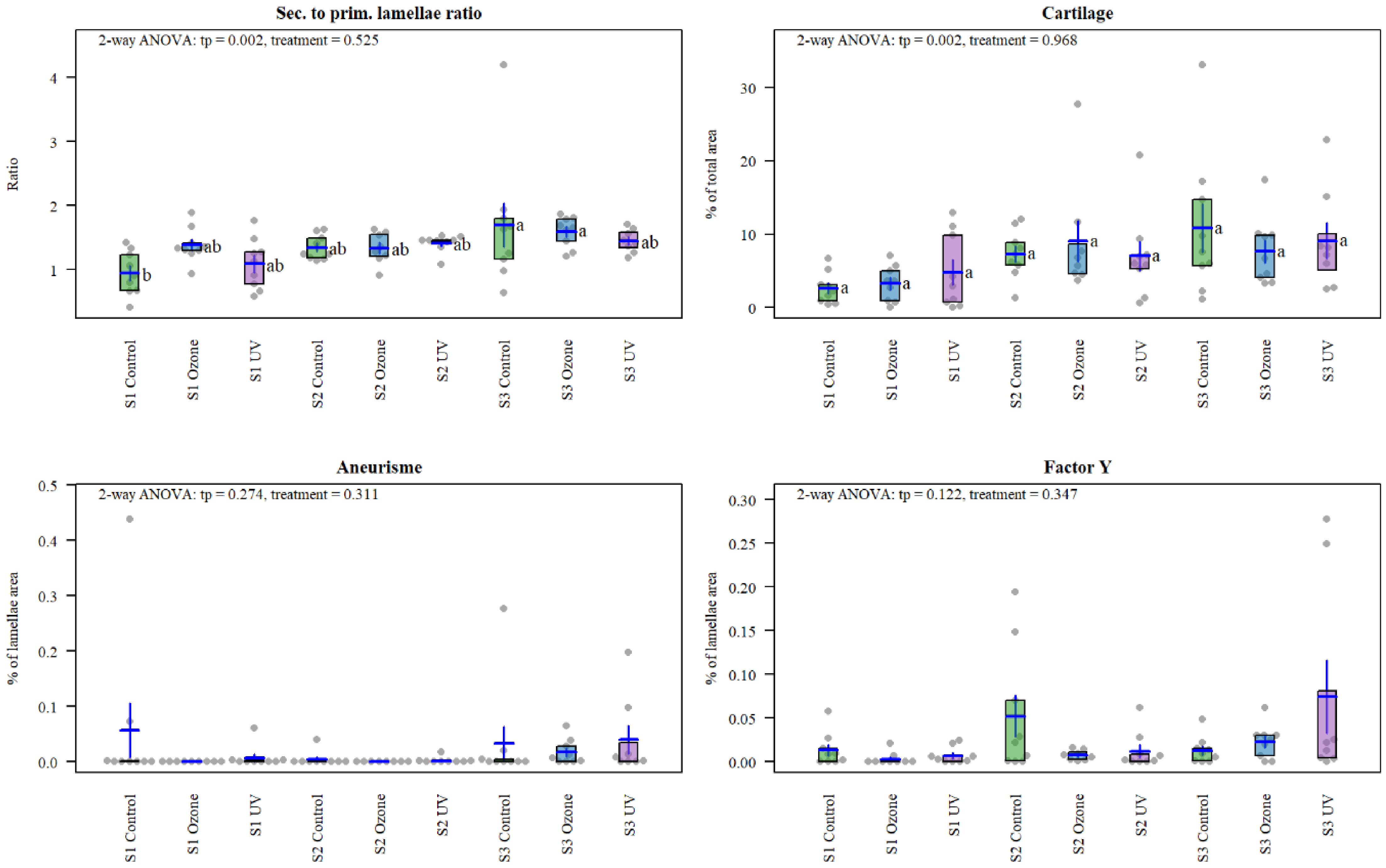
Sec. to prim. lamellae ratio
2-way ANOVA: tp = 0.002, treatment = 0.525
Ratio
b
ab
ab
ab
ab
ab
a
a
ab
Cartilage
2-way ANOVA: tp = 0.002, treatment = 0.968
% of total area
a
Aneurisme
2-way ANOVA: tp = 0.274, treatment = 0.311
% of lamellae area
Factor Y
2-way ANOVA: tp = 0.122, treatment = 0.347
% of lamellae area
S1 Control
S1 Ozone
S1 UV
S2 Control
S2 Ozone
S2 UV
S3 Control
S3 Ozone
S3 UV

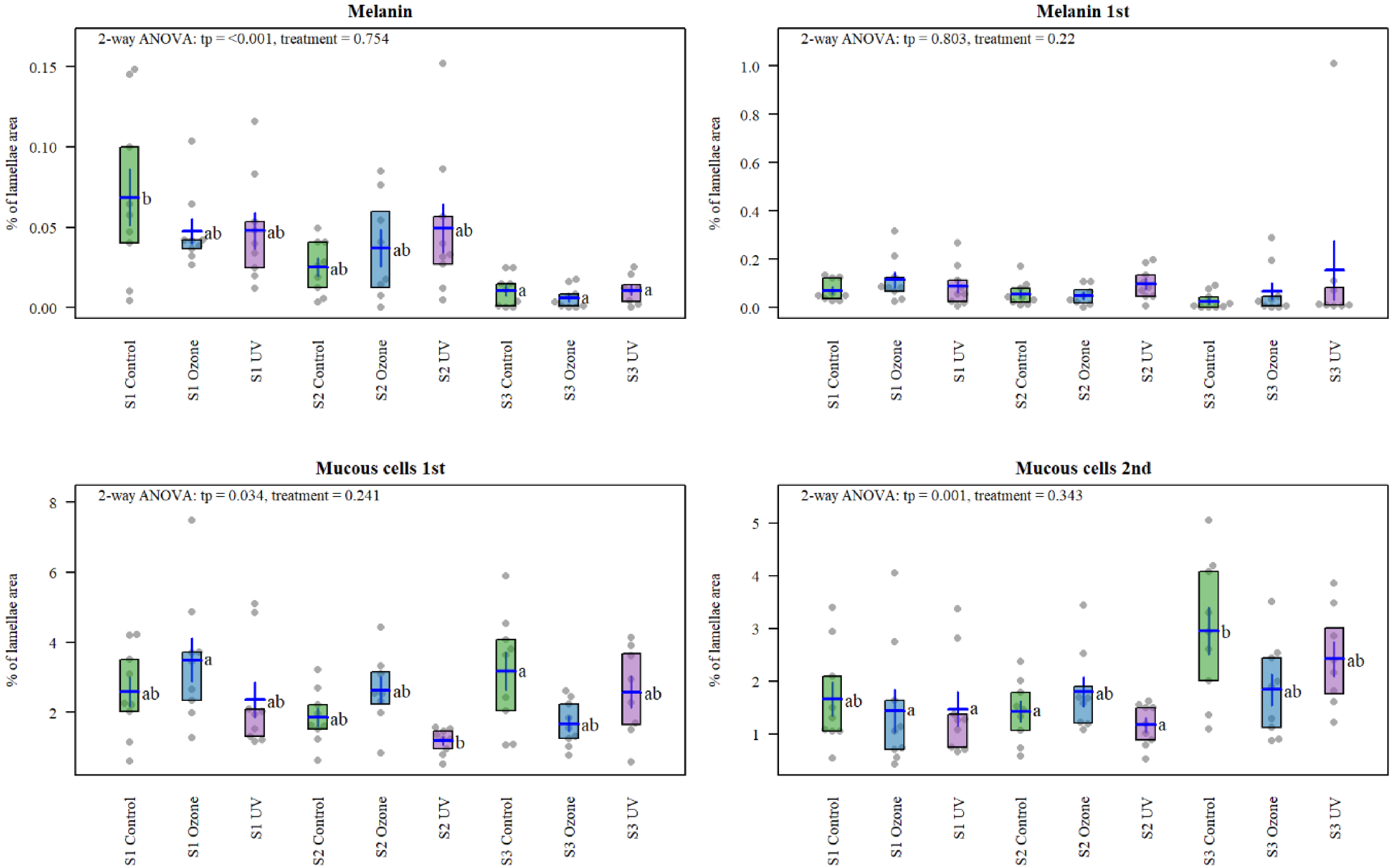
Melanin
2-way ANOVA: tp = <0.001, treatment = 0.754
% of lamellae area
0.00
0.05
0.10
0.15
b
ab
ab
ab
ab
ab
a
a
a
Melanin 1st
2-way ANOVA: tp = 0.803, treatment = 0.22
% of lamellae area
0.0
0.2
0.4
0.6
0.8
1.0
Mucous cells 1st
2-way ANOVA: tp = 0.034, treatment = 0.241
% of lamellae area
2
4
6
8
ab
a
ab
ab
ab
b
a
ab
ab
Mucous cells 2nd
2-way ANOVA: tp = 0.001, treatment = 0.343
% of lamellae area
1
2
3
4
5
ab
a
a
a
ab
a
b
ab
ab
S1 Control
S1 Ozone
S1 UV
S2 Control
S2 Ozone
S2 UV
S3 Control
S3 Ozone
S3 UV

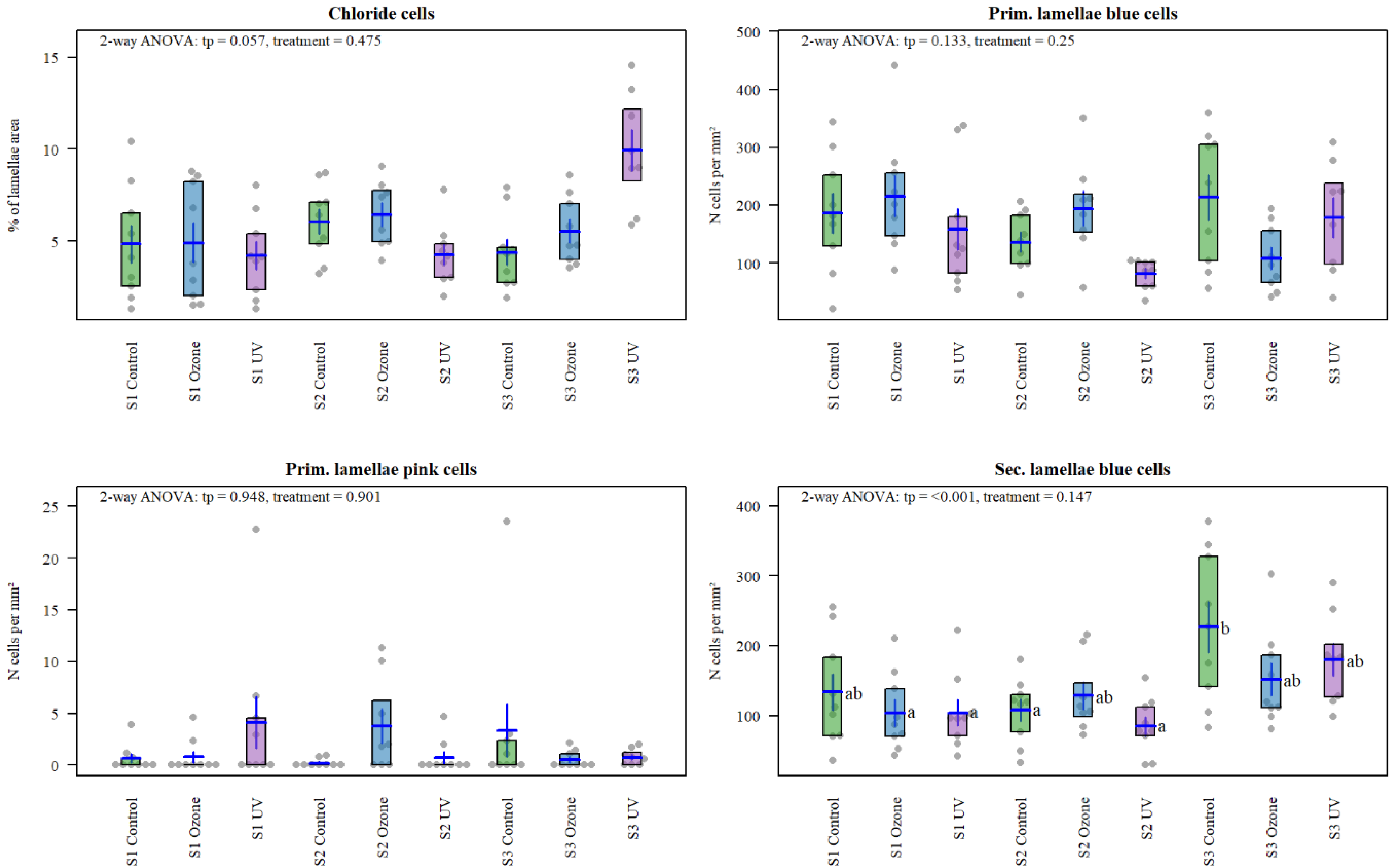

Chloride cells
2-way ANOVA: tp = 0.057, treatment = 0.475
% of lamellae area
15
10
5
Prim. lamellae blue cells
2-way ANOVA: tp = 0.133, treatment = 0.25
N cells per mm²
500
400
300
200
100
Prim. lamellae pink cells
2-way ANOVA: tp = 0.948, treatment = 0.901
N cells per mm²
25
20
15
10
5
0
Sec. lamellae blue cells
2-way ANOVA: tp = <0.001, treatment = 0.147
N cells per mm²
400
300
200
100
ab
a
a
a
ab
a
b
ab
ab
S1 Control
S1 Ozone
S1 UV
S2 Control
S2 Ozone
S2 UV
S3 Control
S3 Ozone
S3 UV

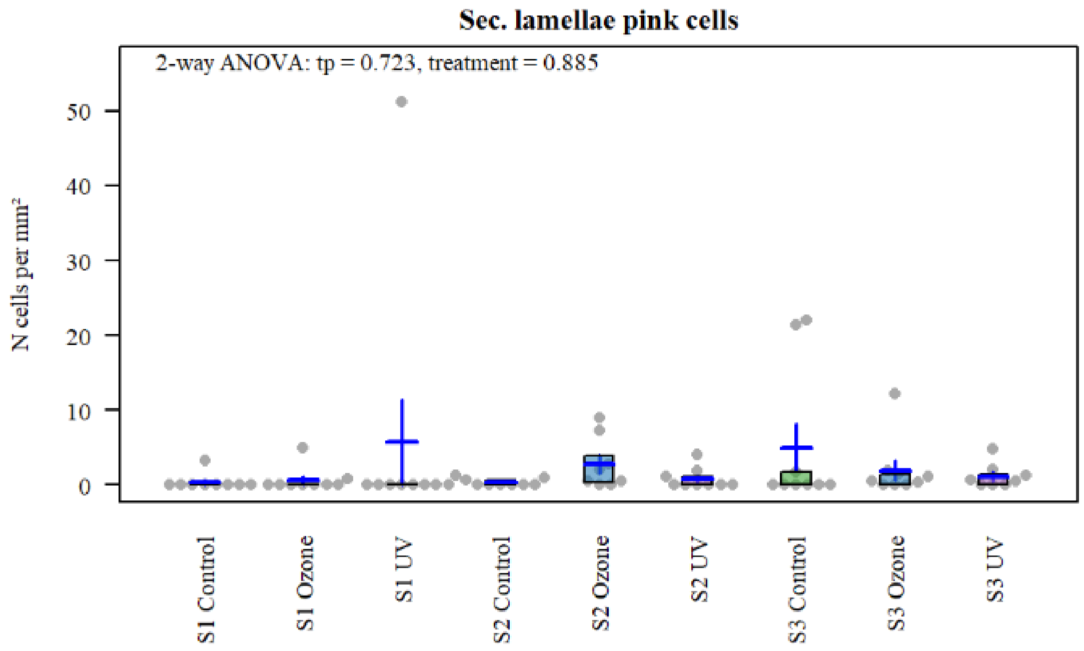
Sec. lamellae pink cells
2-way ANOVA: tp = 0.723, treatment = 0.885
N cells per mm²
0
10
20
30
40
50
S1 Control
S1 Ozone
S1 UV
S2 Control
S2 Ozone
S2 UV
S3 Control
S3 Ozone
S3 UV

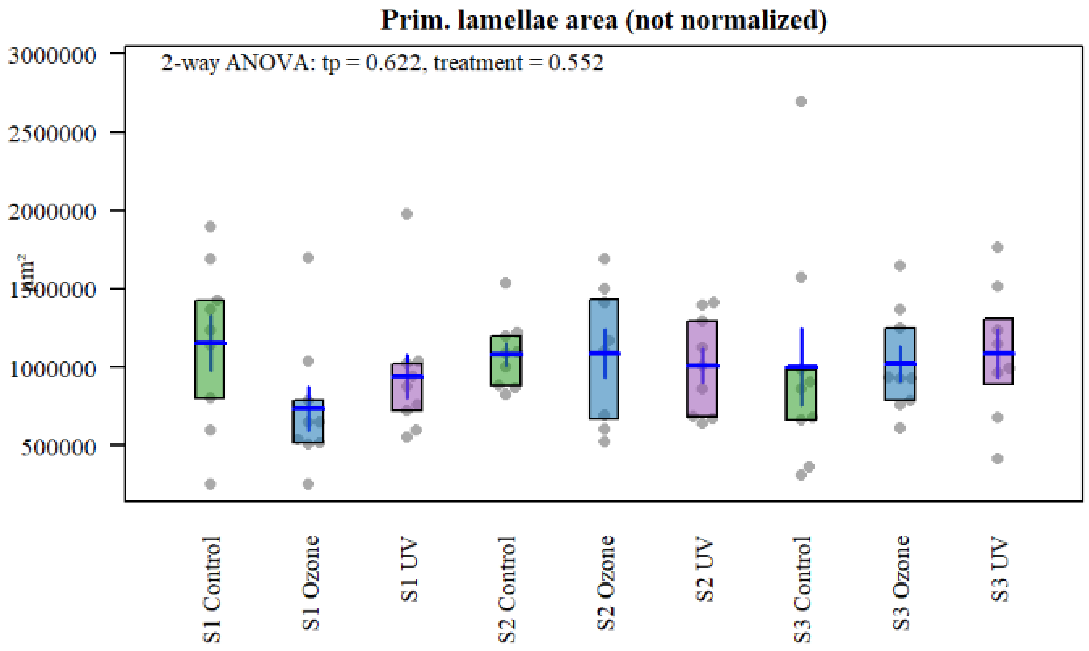
Prim. lamellae area (not normalized)
2-way ANOVA: tp = 0.622, treatment = 0.552
µm²
500000
1000000
1500000
2000000
2500000
3000000
S1 Control
S1 Ozone
S1 UV
S2 Control
S2 Ozone
S2 UV
S3 Control
S3 Ozone
S3 UV

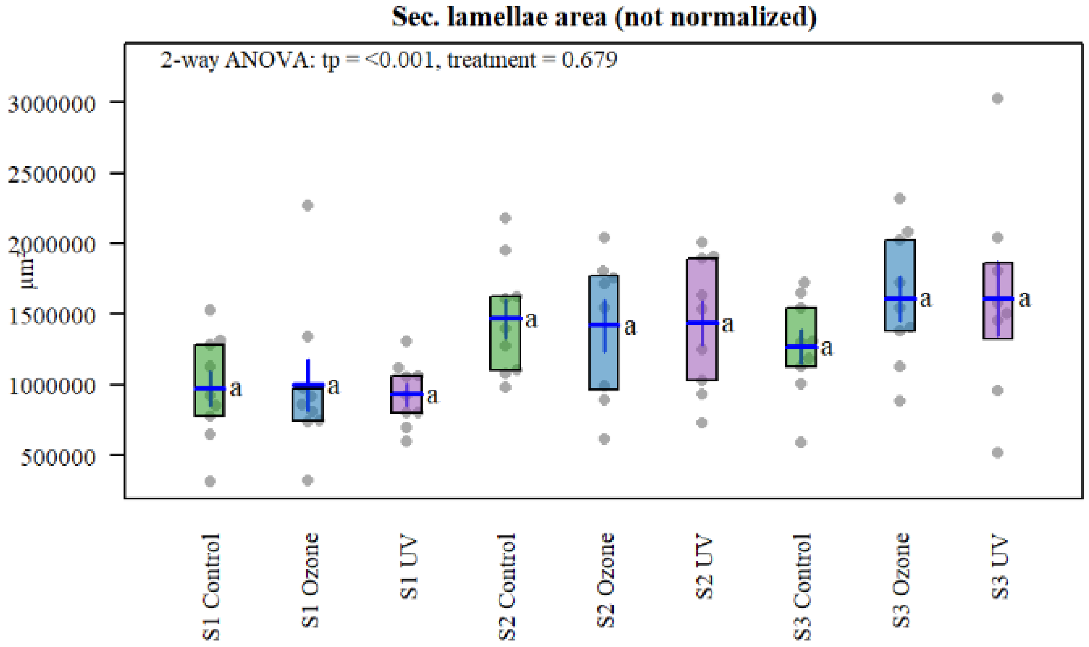
Sec. lamellae area (not normalized)
2-way ANOVA: tp = <0.001, treatment = 0.679
µm²
500000
1000000
1500000
2000000
2500000
3000000
a
S1 Control
S1 Ozone
S1 UV
S2 Control
S2 Ozone
S2 UV
S3 Control
S3 Ozone
S3 UV
