# Supplementary material for: Interplay between disinfection and the enigmatic diplomonad parasite Spironucleus salmonicida in Atlantic salmon
Source: Sci Rep. 2026 May 8;16:21163. doi: 10.1038/s41598-026-51626-4 (PMC13341783; doi:10.1038/s41598-026-51626-4)
Supplement: Supplementary file 5 — Supplementary Material 5 [file 41598_2026_51626_MOESM5_ESM.pdf]

Supplementary File 4

Complete list of parameters histomorphometrically assessed in the skin

A region of interest (ROI) was defined for each sample (one ROI per sample) and analysed using the Aiforia® AI Skin model, which was trained on at least 5768 training regions, derived from a minimum of 125 samples across 24 projects. The skin samples were normalised to the sample's length in millimetres and presented in µm per mm of skin. Values shown represent data from 15 individual fish per sampling point in each treatment group. Statistical significance in each parameter was assessed using two-way ANOVA to evaluate changes within groups over time and differences between groups at specific time points. Significant differences are indicated by different letter annotations, and corresponding p-values are provided.

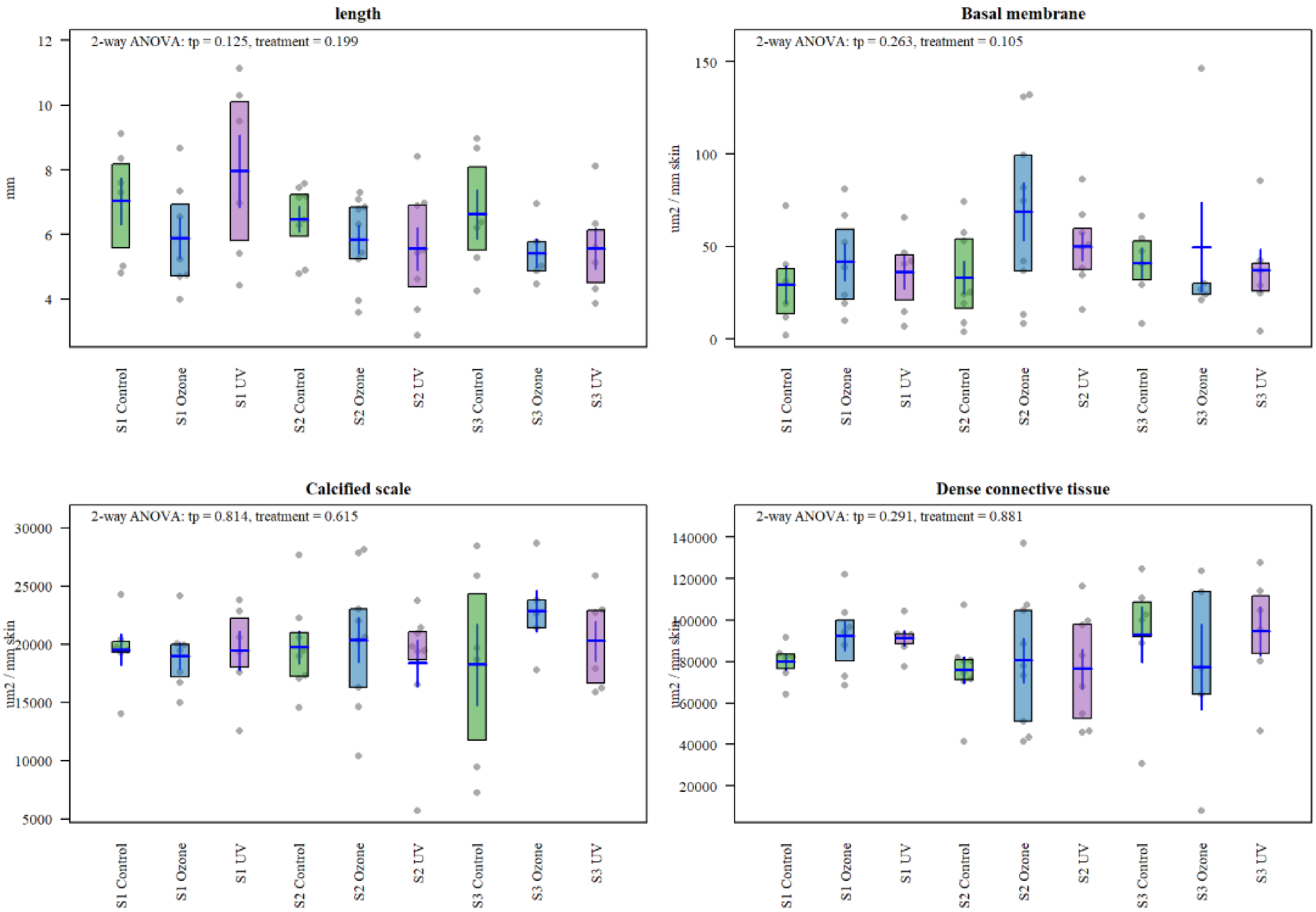

length
2-way ANOVA: tp = 0.125, treatment = 0.199
mm
12
10
8
6
4
Basal membrane
2-way ANOVA: tp = 0.263, treatment = 0.105
um2 / mm skin
150
100
50
0
Calcified scale
2-way ANOVA: tp = 0.814, treatment = 0.615
um2 / mm skin
30000
25000
20000
15000
10000
5000
Dense connective tissue
2-way ANOVA: tp = 0.291, treatment = 0.881
um2 / mm skin
140000
120000
100000
80000
60000
40000
20000
S1 Control
S1 Ozone
S1 UV
S2 Control
S2 Ozone
S2 UV
S3 Control
S3 Ozone
S3 UV

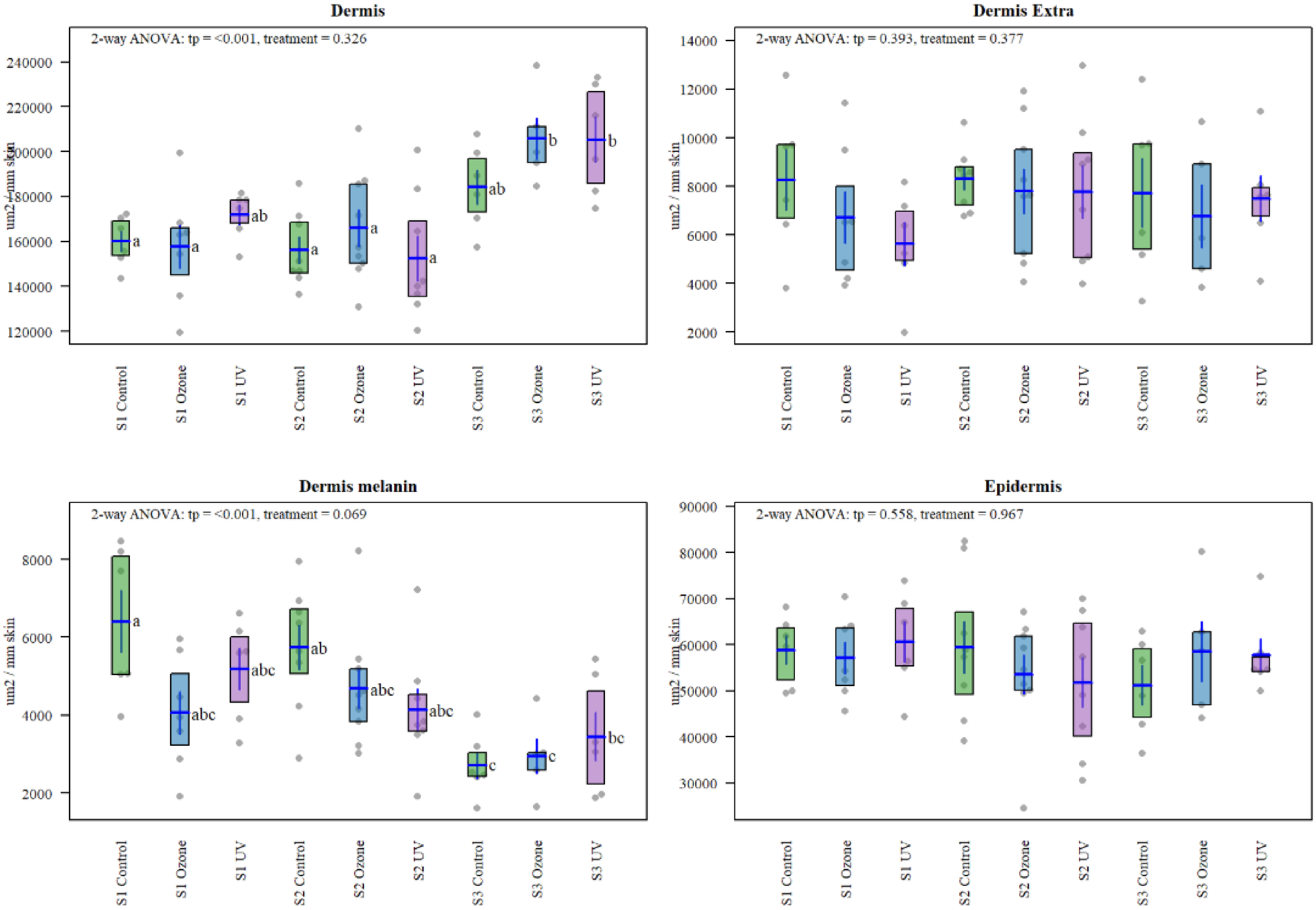
Dermis
2-way ANOVA: tp = <0.001, treatment = 0.326
um2 / mm skin
240000
220000
200000
180000
160000
140000
120000
a
a
ab
a
a
a
ab
b
b
Dermis Extra
2-way ANOVA: tp = 0.393, treatment = 0.377
um2 / mm skin
14000
12000
10000
8000
6000
4000
2000
Dermis melanin
2-way ANOVA: tp = <0.001, treatment = 0.069
um2 / mm skin
8000
6000
4000
2000
a
abc
abc
ab
abc
abc
c
c
bc
Epidermis
2-way ANOVA: tp = 0.558, treatment = 0.967
um2 / mm skin
90000
80000
70000
60000
50000
40000
30000
S1 Control
S1 Ozone
S1 UV
S2 Control
S2 Ozone
S2 UV
S3 Control
S3 Ozone
S3 UV

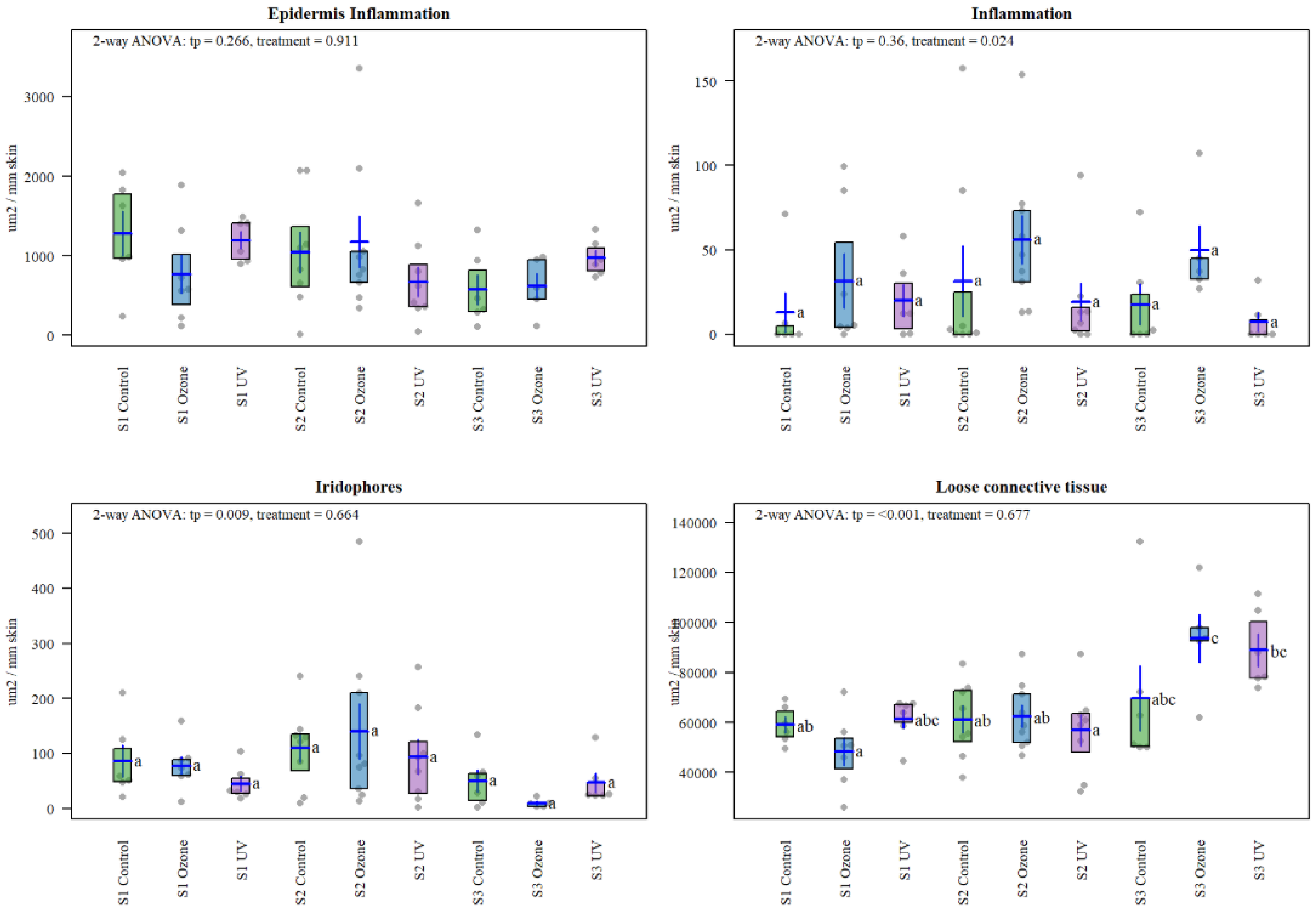
Epidermis Inflammation
2-way ANOVA: tp = 0.266, treatment = 0.911
um2 / mm skin
0
1000
2000
3000
Inflammation
2-way ANOVA: tp = 0.36, treatment = 0.024
0
50
100
150
Iridophores
2-way ANOVA: tp = 0.009, treatment = 0.664
0
100
200
300
400
500
Loose connective tissue
2-way ANOVA: tp = <0.001, treatment = 0.677
40000
60000
80000
100000
120000
140000
a
ab
abc
bc
c
S1 Control
S1 Ozone
S1 UV
S2 Control
S2 Ozone
S2 UV
S3 Control
S3 Ozone
S3 UV

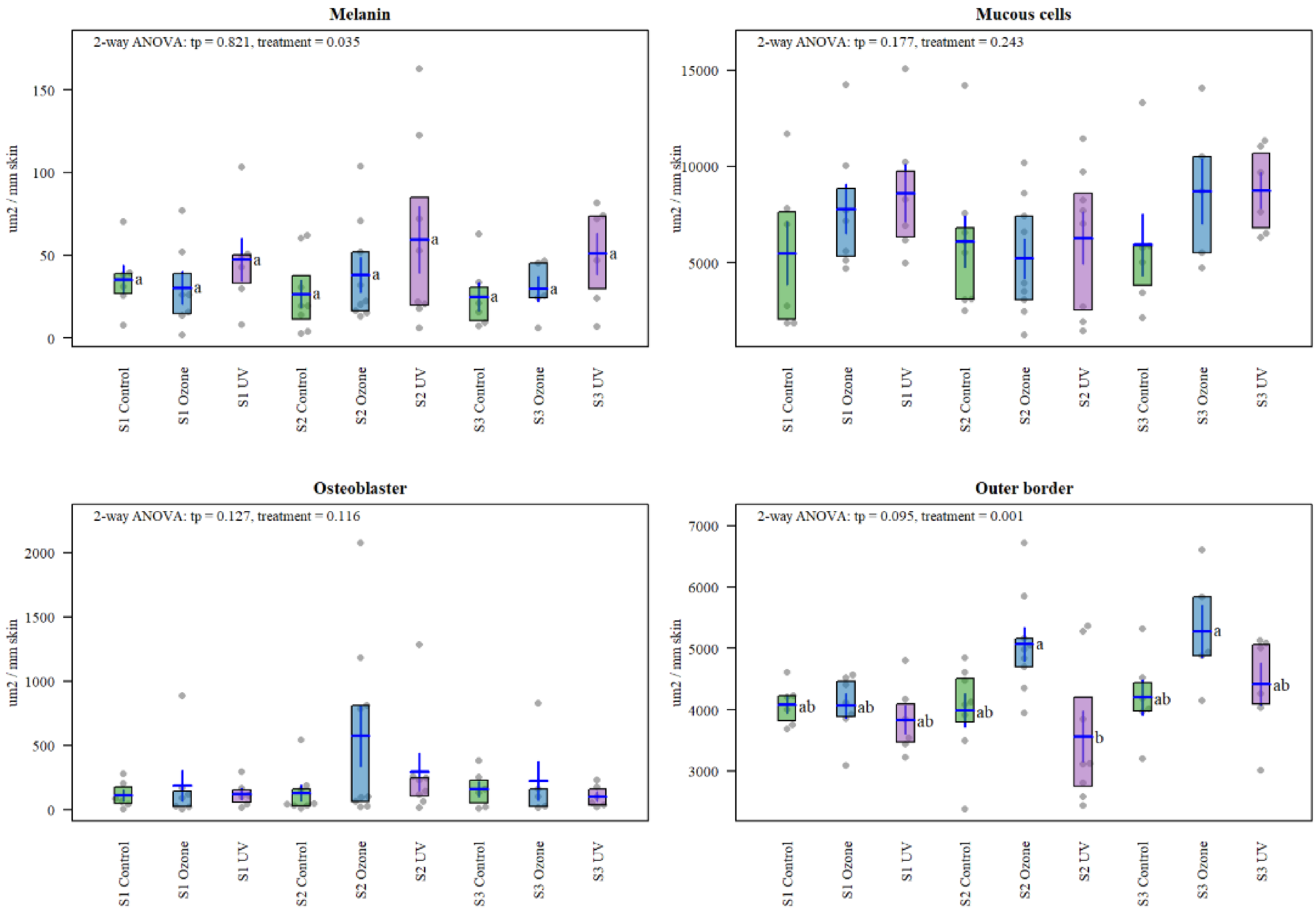
Melanin
2-way ANOVA: tp = 0.821, treatment = 0.035
um2 / mm skin
0
50
100
150
a
S1 Control
S1 Ozone
S1 UV
S2 Control
S2 Ozone
S2 UV
S3 Control
S3 Ozone
S3 UV
Mucous cells
2-way ANOVA: tp = 0.177, treatment = 0.243
um2 / mm skin
5000
10000
15000
S1 Control
S1 Ozone
S1 UV
S2 Control
S2 Ozone
S2 UV
S3 Control
S3 Ozone
S3 UV
Osteoblaster
2-way ANOVA: tp = 0.127, treatment = 0.116
um2 / mm skin
0
500
1000
1500
2000
S1 Control
S1 Ozone
S1 UV
S2 Control
S2 Ozone
S2 UV
S3 Control
S3 Ozone
S3 UV
Outer border
2-way ANOVA: tp = 0.095, treatment = 0.001
um2 / mm skin
3000
4000
5000
6000
7000
ab
a
b
S1 Control
S1 Ozone
S1 UV
S2 Control
S2 Ozone
S2 UV
S3 Control
S3 Ozone
S3 UV

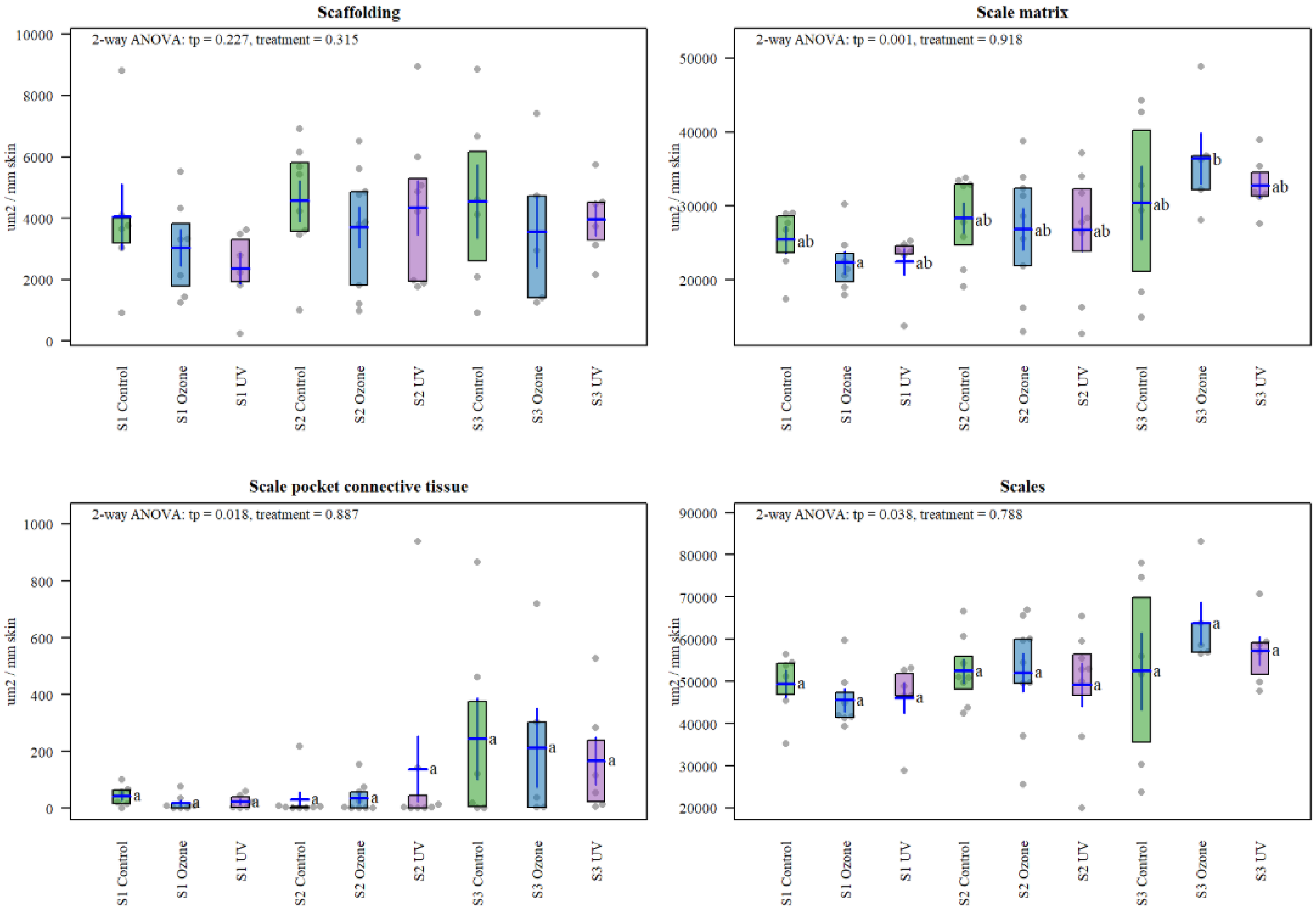
Scaffolding
2-way ANOVA: tp = 0.227, treatment = 0.315
um2 / mm skin
0
2000
4000
6000
8000
10000
Scale matrix
2-way ANOVA: tp = 0.001, treatment = 0.918
um2 / mm skin
20000
30000
40000
50000
ab
a
ab
ab
ab
ab
ab
b
ab
Scale pocket connective tissue
2-way ANOVA: tp = 0.018, treatment = 0.887
um2 / mm skin
0
200
400
600
800
1000
a
a
a
a
a
a
a
a
a
Scales
2-way ANOVA: tp = 0.038, treatment = 0.788
um2 / mm skin
20000
30000
40000
50000
60000
70000
80000
90000
a
a
a
a
a
a
a
a
a
S1 Control
S1 Ozone
S1 UV
S2 Control
S2 Ozone
S2 UV
S3 Control
S3 Ozone
S3 UV

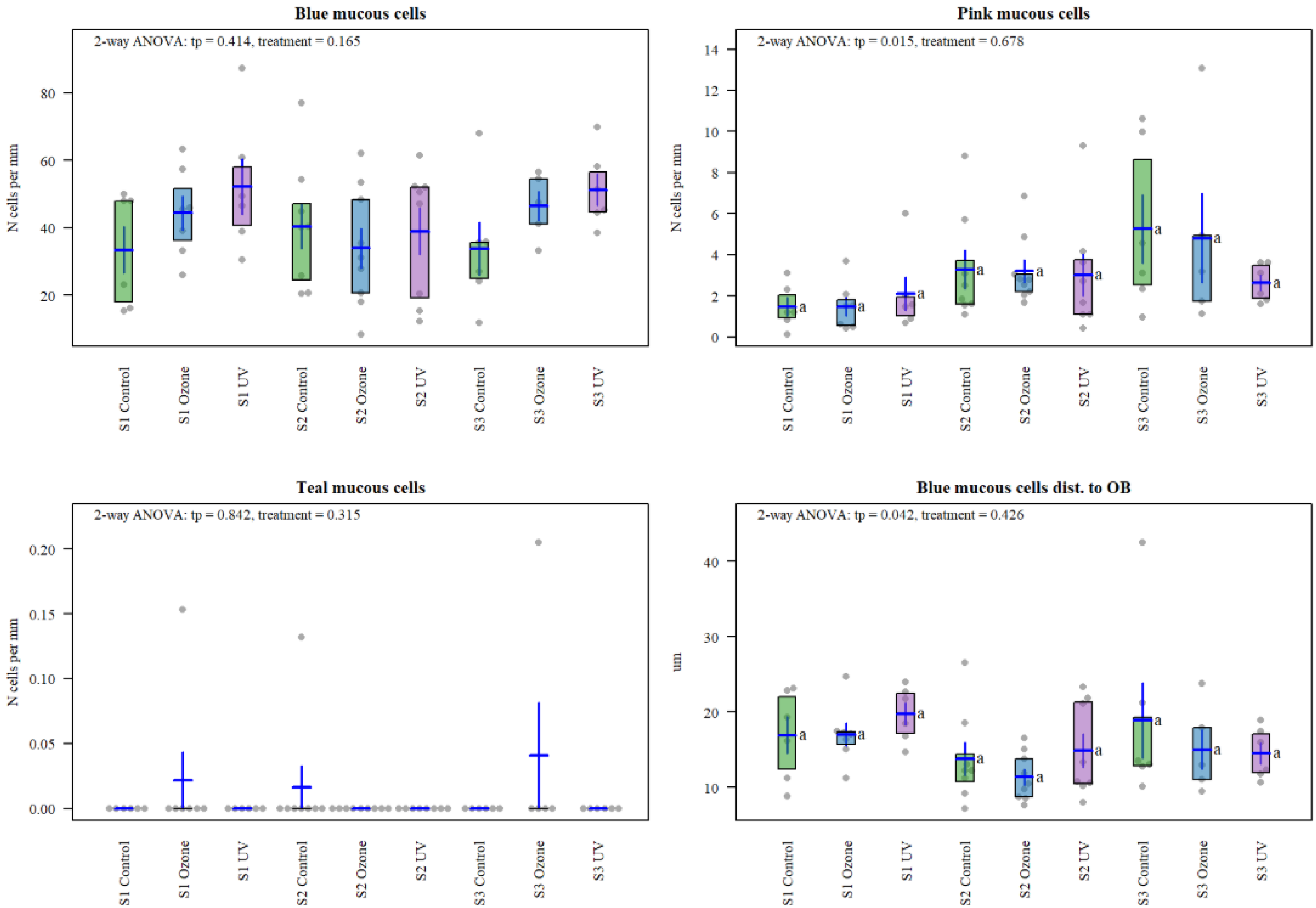
Blue mucous cells
2-way ANOVA: tp = 0.414, treatment = 0.165
N cells per mm
20
40
60
80
Pink mucous cells
2-way ANOVA: tp = 0.015, treatment = 0.678
N cells per mm
0
2
4
6
8
10
12
14
Teal mucous cells
2-way ANOVA: tp = 0.842, treatment = 0.315
N cells per mm
0.00
0.05
0.10
0.15
0.20
Blue mucous cells dist. to OB
2-way ANOVA: tp = 0.042, treatment = 0.426
um
10
20
30
40
S1 Control
S1 Ozone
S1 UV
S2 Control
S2 Ozone
S2 UV
S3 Control
S3 Ozone
S3 UV

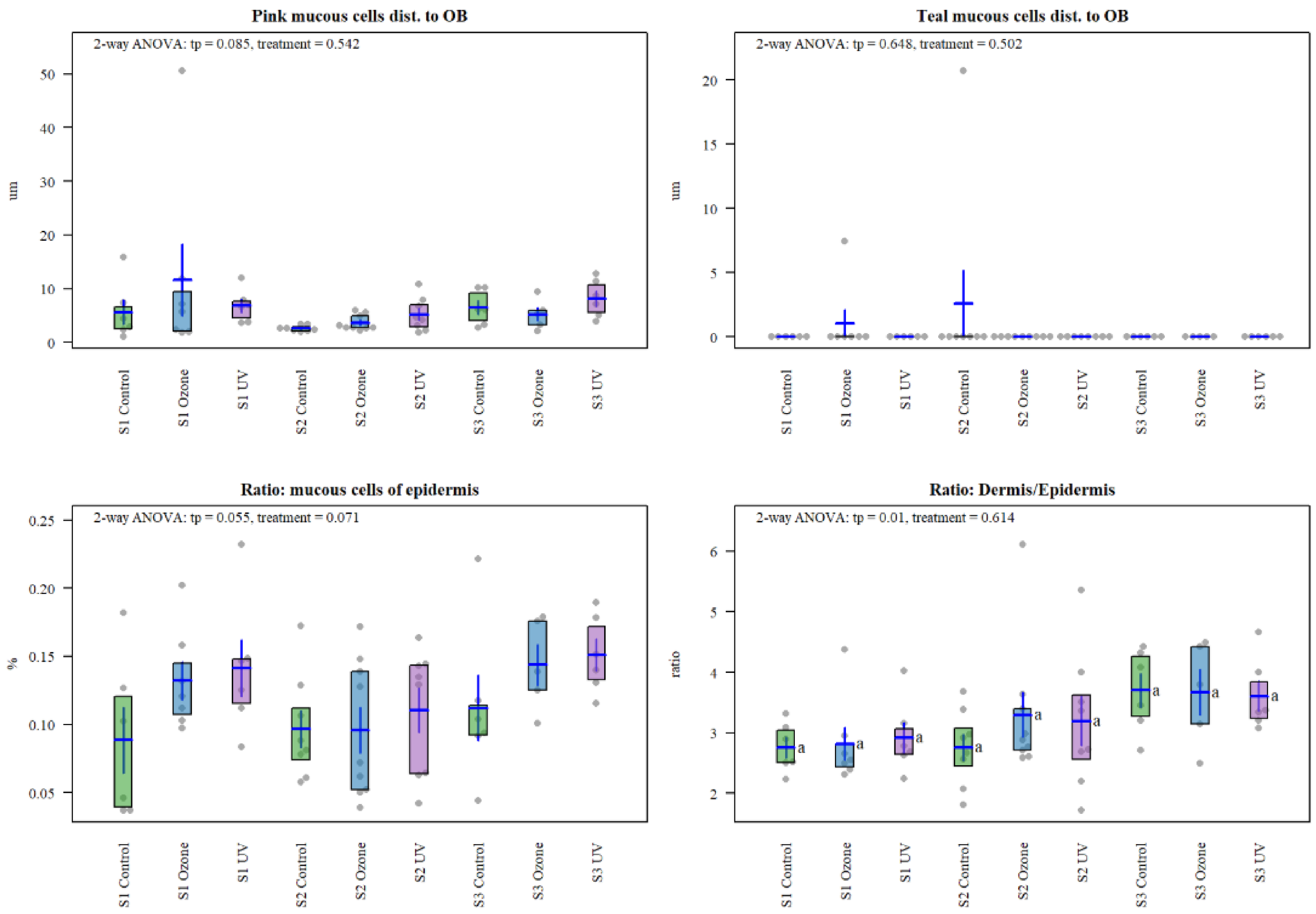

Pink mucous cells dist. to OB
2-way ANOVA: tp = 0.085, treatment = 0.542
um
0
10
20
30
40
50
Teal mucous cells dist. to OB
2-way ANOVA: tp = 0.648, treatment = 0.502
um
0
5
10
15
20
Ratio: mucous cells of epidermis
2-way ANOVA: tp = 0.055, treatment = 0.071
%
0.05
0.10
0.15
0.20
0.25
Ratio: Dermis/Epidermis
2-way ANOVA: tp = 0.01, treatment = 0.614
ratio
2
3
4
5
6
a
S1 Control
S1 Ozone
S1 UV
S2 Control
S2 Ozone
S2 UV
S3 Control
S3 Ozone
S3 UV
